# Dissection of brain-wide resting-state and functional somatosensory circuits by fMRI with optogenetic silencing

**DOI:** 10.1073/pnas.2113313119

**Published:** 2022-01-18

**Authors:** Won Beom Jung, Haiyan Jiang, Soohyun Lee, Seong-Gi Kim

**Affiliations:** ^a^Center for Neuroscience Imaging Research, Institute for Basic Science, Suwon 16419, Republic of Korea;; ^b^Department of Biomedical Engineering, Sungkyunkwan University, Suwon 16419, Republic of Korea;; ^c^Unit on Functional Neural Circuits, NIH, Bethesda, MD 20892;; ^d^Department of Intelligent Precision Healthcare Convergence, Sungkyunkwan University, Suwon 16419, Republic of Korea

**Keywords:** fMRI, somatosensory network, neuronal inhibition, circuit dissection, resting-state connectivity

## Abstract

To further advance functional MRI (fMRI)–based brain science, it is critical to dissect fMRI activity at the circuit level. To achieve this goal, we combined brain-wide fMRI with neuronal silencing in well-defined regions. Since focal inactivation suppresses excitatory output to downstream pathways, intact input and suppressed output circuits can be separated. Highly specific cerebral blood volume–weighted fMRI was performed with optogenetic stimulation of local GABAergic neurons in mouse somatosensory regions. Brain-wide spontaneous somatosensory networks were found mostly in ipsilateral cortical and subcortical areas, which differed from the bilateral homotopic connections commonly observed in resting-state fMRI data. The evoked fMRI responses to somatosensory stimulation in regions of the somatosensory network were successfully dissected, allowing the relative contributions of spinothalamic (ST), thalamocortical (TC), corticothalamic (CT), corticocortical (CC) inputs, and local intracortical circuits to be determined. The ventral posterior thalamic nucleus receives ST inputs, while the posterior medial thalamic nucleus receives CT inputs from the primary somatosensory cortex (S1) with TC inputs. The secondary somatosensory cortex (S2) receives mostly direct CC inputs from S1 and a few TC inputs from the ventral posterolateral nucleus. The TC and CC input layers in cortical regions were identified by laminar-specific fMRI responses with a full width at half maximum of <150 µm. Long-range synaptic inputs in cortical areas were amplified approximately twofold by local intracortical circuits, which is consistent with electrophysiological recordings. Overall, whole-brain fMRI with optogenetic inactivation revealed brain-wide, population-based, long-range circuits, which could complement data typically collected in conventional microscopic functional circuit studies.

## Significance

Functional MRI (fMRI) has led to tremendous advancements in brain science by allowing noninvasive mapping of functional regions in response to various stimuli and noninvasive mapping of resting-state functional connectivity. Both evoked and resting-state functional networks contain multiple brain regions that are hierarchically yet reciprocally connected. Therefore, it is critical to determine the relative contributions of different circuits to fMRI findings to better understand brain functions and resting-state connectivity. Here, we adopted local silencing with optogenetic stimulation to suppress downstream networks and successfully dissected fMRI responses at the circuit level. This fMRI approach opens an avenue for understanding brain-wide, population-based neural circuits, allowing investigations of functional reorganization caused by neuropathological modifications and learning in individual animals.

Functional MRI (fMRI) has led to tremendous advances in brain science by enabling noninvasive mapping of both functional regions with various stimuli and resting-state functional connectivity. Evoked fMRI can be used to detect the strength of hemodynamic responses to external stimuli (1–3), whereas resting-state fMRI (rs-fMRI) measures the degree of synchrony between fMRI time series among anatomically distinct brain regions at rest (4–7). Both evoked and resting-state functional networks contain multiple brain regions that are hierarchically yet reciprocally connected by ascending thalamocortical (TC), descending corticothalamic (CT), corticocortical (CC), and intracortical (IC) circuits (8). Therefore, it is critical to determine the relative contributions of different circuits to fMRI findings to better understand brain functions and resting-state connectivity.

To identify the contributions of neural networks to fMRI responses, we aimed to silence neural activity in well-defined regions via temporally specific optogenetic control of a given neural population (9). Inhibiting one cortical region inevitably suppresses excitatory output to downstream signaling pathways (e.g., CT, CC, and IC pathways) (10–12), and the down-regulated neuronal activity reflects the degree of interregional communication under basal conditions (12). Therefore, fMRI with cortical inactivation is beneficial for brain-wide mapping of functional connectivity in the resting state. Similarly, local silencing during exposure to external stimuli suppresses downstream activity (output-related circuits) without compromising the upstream and/or collateral inputs from other brain regions (input-related circuits) (11, 13–16); thus, downstream circuit contributions to fMRI can be determined by comparing evoked fMRI responses with and without focal inactivation.

In the current study, we adopted a widely investigated somatosensory network involving multiple brain regions, such as the first-order ventral posterior thalamic nucleus, higher-order posterior medial thalamic nucleus (POm), primary somatosensory cortex (S1), secondary somatosensory cortex (S2), and primary motor cortex (M1) (17, 18). Anatomical tracing studies have demonstrated the existence of complex monosynaptic networks across broad somatosensory-related brain areas (19–21). In addition, microscopic functional circuits in preselected areas have been delineated using electrophysiology (22–26) and optical recordings (27, 28) combined with optogenetic and chemogenetic tools. Recently, macroscopic functional activity in the whole brain was mapped by fMRI (29–32). Thus, the somatosensory system is an ideal model for investigating whether the contributions of different projections to fMRI responses can be separated.

To investigate spontaneous and sensory-evoked fMRI signals at the circuit level, we combined highly specific cerebral blood volume (CBV)–weighted fMRI at an ultra-high magnetic field strength of 15.2 T with optogenetic stimulation of local GABAergic neurons using the vesicular GABA transporter (VGAT)-channelrhodopsin-2 (ChR2)–enhanced yellow fluorescent protein (EYFP) transgenic mouse line (33). We examined how different cortical areas, including the primary somatosensory forelimb (S1FL), M1, and S2, affect activity in cortical and subcortical brain regions to determine causal relationships in the information flow among multiple brain areas.

## Results

**Fig. 1.**
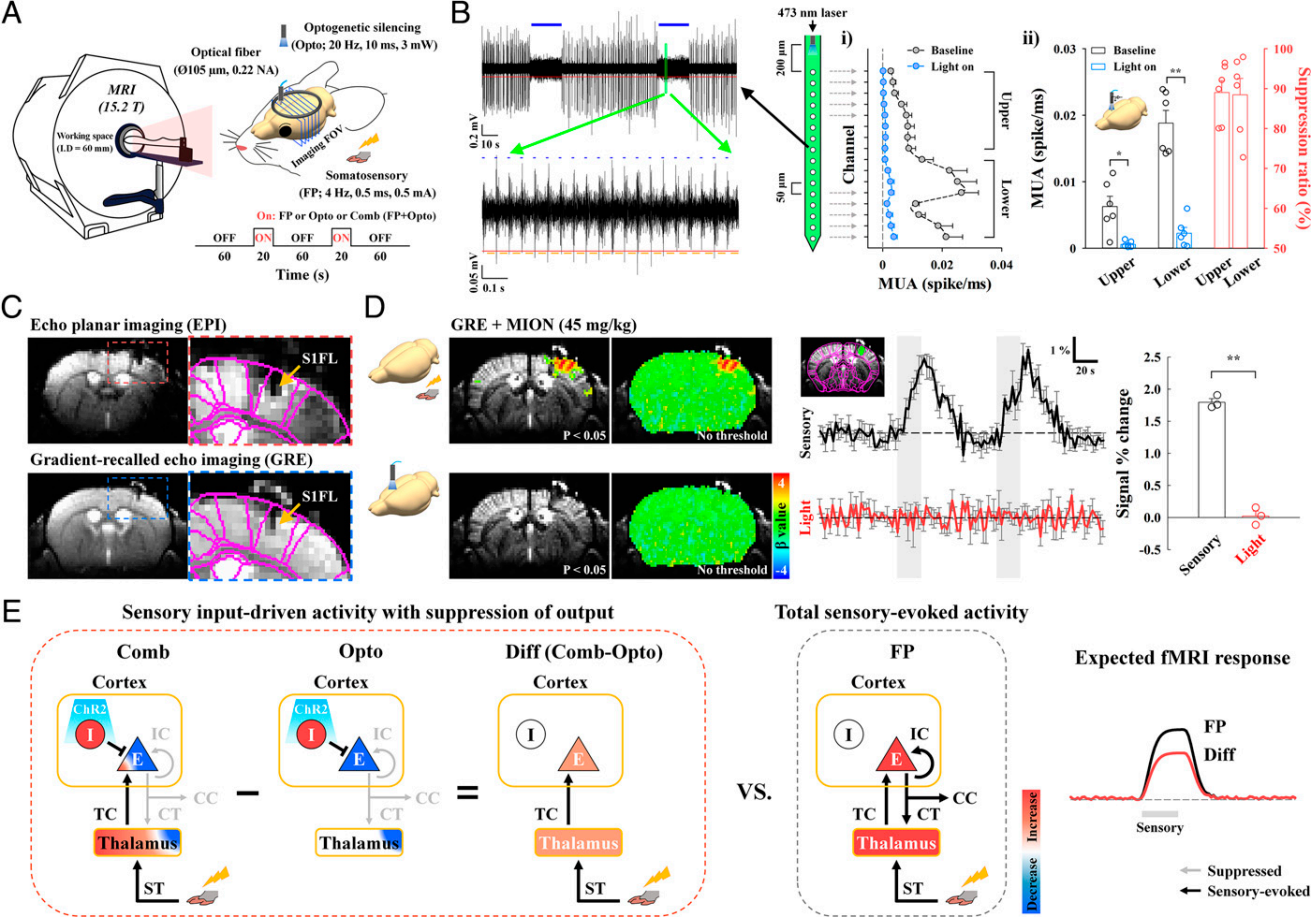
Experimental setup for high-resolution, CBV-weighted optogenetic mouse fMRI and conceptual neural circuit analysis. (*A*) Schematic of multislice fMRI at 15.2 T during FP somatosensory stimulation and during cortical inactivation without (Opto) and with (Comb) somatosensory stimulation. (*B*) Silencing spontaneous activity of excitatory neurons in the S1FL by optogenetic excitation of inhibitory neurons in VGAT-ChR2 mice (*n* = 6). A 16-channel optoelectrode with 50 µm interchannel spacing was inserted to a 1-mm depth, and photostimulation was delivered on the surface of the cortex. During two 20-s photostimulation periods (blue bars in the left example recording trace), MUAs were reduced; in the expanded view, MUA is shown to slightly increased during each pulse (blue dots) due to enhanced inhibitory activity but was near zero between pulses (yellowish orange lines) due to suppressed spontaneous activity. During 20 s of photostimulation, spontaneous activity in i) all cortical layers (200- to 1,000-µm depth) and ii) both the upper and lower eight channels was mostly suppressed by activation of inhibitory neurons. Similar uniform suppression across all cortical depths was achieved during photostimulation at the middle of the cortex (*SI Appendix*, Fig. S2*B*). Blue horizontal bar, 20-s optogenetic stimulus; red, MUA amplitude threshold; yellowish orange, spontaneous MUA count duration; error bar, SEM; *P < 0.05 and **P < 0.01 (paired *t* test); and suppression ratio in panel ii, attenuation rate of neural activity during optogenetic silencing relative to the baseline activity. (*C*) High-resolution MRI images of the brain with an optical fiber targeting the S1FL. The image artifacts of distortion and signal drops caused by the optical fiber in gradient-echo echo planar imaging (EPI) (red-dashed box) were minimized by the adoption of gradient-echo imaging with a short TE of 3 ms (yellow arrow, fiber position). For the CBV-weighted fMRI study, a 45-mg/kg dose of a superparamagnetic monocrystalline iron oxide nanoparticle (MION) agent was injected into the animals’ blood. S1FL, primary somatosensory area of the forelimb. Images of a fiber position and a choice of the MION dose are shown in *SI Appendix*, Fig. S3. (*D*) No artifactual fMRI responses to light stimulation were observed in naïve mice. To assess potential light-induced MRI artifacts, functional studies in naïve mice were conducted with interleaved FP stimulation and photostimulation of the S1FL with the same experimental protocol used for fMRI with cortical inactivation (*n* = 3). Somatosensory fMRI was used as the internal control to ensure the reliability of fMRI responses to external stimuli. Sensory-evoked fMRI responses were observed in the S1FL, but responses during photostimulation were absent in fMRI maps with and without a statistical threshold (uncorrected *P* < 0.05) and in time courses of the S1FL. Thus, fMRI signal changes resulting from light-induced tissue heating were negligible. Black and red time traces, somatosensory-evoked and photostimulated fMRI time courses in the S1FL, respectively; gray vertical bar in time courses, 20-s stimulus; error bars, SEM; and **P < 0.01 (paired *t* test). (*E*) Schematic diagrams to dissect sensory-evoked, long-range, and local activity by fMRI with the assistance of optogenetic cortical inhibition. In a simplified circuit, excitatory neurons (E) interacting with inhibitory interneurons (I) in the cortex received TC inputs from the thalamus and produced spiking outputs for IC, CC, and CT downstream activity. Silencing cortical excitatory neurons by activating ChR2-expressing cortical inhibitory neurons suppressed outputs to downstream CT, CC, and IC pathways (Opto), while simultaneous FP stimulation induced sensory-evoked upstream ST and TC inputs to the thalamus and cortex, respectively (Comb). After subtracting fMRI responses to optogenetic inactivation without FP stimulation (Opto) from those with FP stimulation (Comb), the difference signal was related to upstream, input-driven responses (Diff; Comb-Opto) without the direct contribution of inhibitory neural activity. The relative contribution of downstream CT, CC, and IC pathways can be determined from the total sensory-evoked activity (Diff versus FP). Blue-to-red color scale, decrease-to-increase relative to baseline activity.

To map brain-wide spontaneous and evoked long-range somatosensory networks by fMRI, VGAT-ChR2-EYFP mice expressing light-sensitive opsin proteins in GABAergic interneuron populations were used (refer to *SI Appendix*, Fig. S1 for expression in inhibitory neurons). An optical fiber cannula (105-µm inner core diameter) was implanted into the middle of the cortex in the S1FL or S2 and the upper cortical area in M1 (*SI Appendix*, Fig. S2*A*) to silence local pyramidal neuronal activities via optogenetic stimulation of inhibitory neurons with blue light (473 nm, 3 mW, 10-ms duration, and 20 Hz) (Fig. 1*A*).

The suppression of neural activity was confirmed by recording multiunit activities (MUAs) with 16-channel optoelectrode under the same photostimulation conditions used for fMRI studies. During 20 s of photostimulation to the cortical surface of the S1FL, an increase in stimulation-induced inhibitory neuronal activity mostly suppressed spontaneous activity across all cortical depths (Fig. 1*B*; baseline [black profile and bar] versus light on [blue profile and bar]; *n* = 6 mice), which is consistent with previous findings (33, 34). Similar uniform suppression was observed across all cortical depths when photostimulation was targeted at a depth of 500 µm in the S1FL (*SI Appendix*, Fig. S2*B*). With 3-mW optogenetic stimulation of VGAT-ChR2, the spatial spread of inhibition was expected to be >1 mm from the center of fiber (34), which covers the S1FL, M1, or S2 areas.

Optogenetic fMRI of anesthetized VGAT-ChR2 mice was performed with an ultra-high magnetic field of 15.2 T. Fiber implantation often causes image distortions and signal dropouts in echo planar imaging scans commonly used for fMRI studies, which are more severe with higher magnetic fields (refer to red box of Fig. 1*C* and *SI Appendix*, Fig. S3*A*). Thus, we adopted a conventional gradient-echo imaging technique with a short echo time (TE) of 3 ms to minimize TE-sensitive blood oxygenation level–dependent (BOLD) contributions and image distortions. This approach allowed us to identify fiber positions (refer to blue box of Fig. 1*C* and *SI Appendix*, Fig. S3*A*) and coregister brain MRI scans with the mouse brain atlas. To enhance functional sensitivity and specificity, 45 mg/kg superparamagnetic iron oxide nanoparticles were injected into the blood of the animals (reference *SI Appendix*, Fig. S3*B* for selection of the iron oxide dose), which resulted in CBV sensitization. An increase in CBV increased the amount of iron oxide within the voxel, thus decreasing fMRI signals. Therefore, the change in the polarity of the original CBV-weighted fMRI signal was inverted to match that of the CBV responses. Multislice CBV-weighted fMRI scans were obtained with spatial resolution = 156 × 156 × 500 μm^3^ and temporal resolution = 2 s for mapping functional connectivity with highly specific blood volume responses. Photostimulation did not induce heating-related fMRI artifacts in naïve mice (Fig. 1*D*; C57BL/6J, *n* = 3 mice); thus, fMRI responses to optogenetic stimulation genuinely reflected underlying neural activity.

To dissect the functional circuits by fMRI, optogenetic cortical inhibition was adopted during somatosensory stimulation (Fig. 1*E*). Silencing cortical excitatory neurons by optogenetically activating ChR2-expressing inhibitory neurons suppresses local excitatory recurrent circuits and outputs to downstream pathways (15, 35) (Opto in Fig. 1*E*), resulting in a decreased fMRI signal in local and downstream networked areas (deactivation). Notably, most cortical GABAergic neurons do not project other brain regions. Therefore, this approach can be used to map spontaneous neural interactions at rest. Simultaneous forepaw (FP) stimulation during cortical silencing can preserve sensory-evoked inputs to the thalamus and cortex via upstream spinothalamic (ST) and TC pathways with downstream suppression (Comb in Fig. 1*E*). Since inhibitory neural activation induces a hemodynamic response at the stimulation site (36, 37) and in downstream regions, the contribution by this type of activation should be removed. It is assumed that the common photostimulation-driven hemodynamic response is completely eliminated by calculating the difference between the optogenetic fMRI responses observed with and without sensory stimulation (Comb − Opto = Diff in Fig. 1*E*). Therefore, the remaining signal is due to the contribution of upstream ST and TC pathways in the absence of local recurrent circuits and downstream projections from a given target region. Then, the contribution of downstream suppression of fMRI responses can be determined from somatosensory-evoked fMRI responses (Diff versus FP somatosensory stimulation in Fig. 1*E*), allowing us to determine the functional causality for long-range and local processing in somatosensory networks. Although a similar circuit-analysis approach of sensory stimulation with and without local silencing has been used for electrophysiological studies in preselected areas (13–16), fMRI with and without optogenetic inhibition can be used to determine brain-wide long-range circuits at a population level.

### Mapping Somatosensory Networks of Resting-State Activity: rs-fMRI versus Cortical Silencing fMRI.

**Fig. 2.**
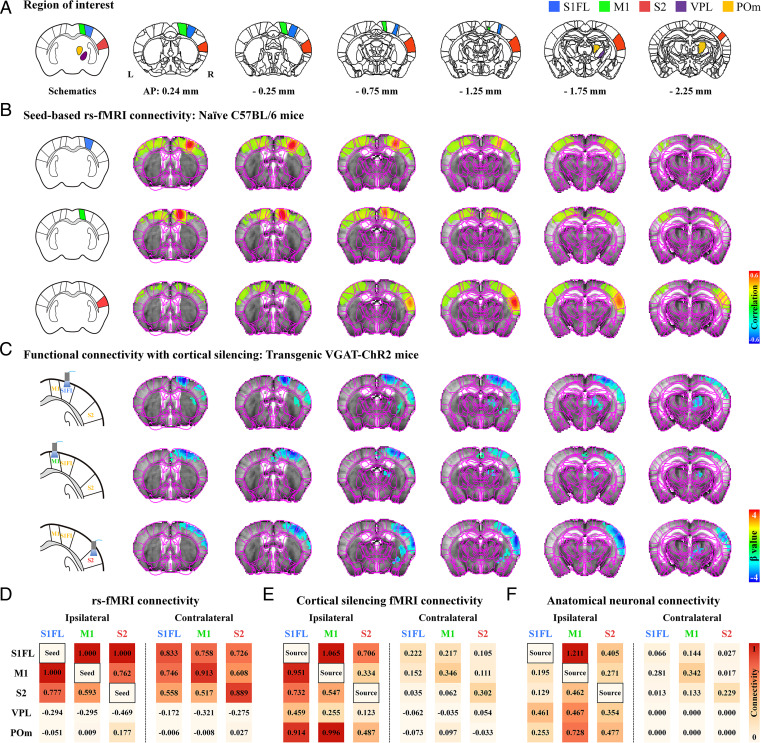
The spontaneous resting-state somatosensory network with optogenetic neuronal inactivation differs from conventional resting-state fMRI connectivity. (*A*) Allen Mouse Brain Atlas–based anatomic ROIs in the somatosensory network were chosen for analyzing resting-state connectivity. (*B* and *C*) Brain-wide functional connectivity maps of spontaneous activity measured by seed-based rs-fMRI and fMRI with cortical inactivation (with Fig. 1*A* Opto paradigm). Brain atlas drawings (leftmost images) show seed ROIs and optogenetic stimulation sites. (*B*) Strong bilateral homotopic correlations were observed in seed-based rs-fMRI for the S1FL, M1, and S2 (wild-type, *n* = 5) and in seed-based rs-fMRI of transgenic VGAT-ChR2 mice (*SI Appendix*, Fig. S4*A*). However, ipsilateral somatosensory regions (*C*), including the cortices and thalamic nuclei, mostly responded to optogenetic cortical inactivation of the S1FL (VGAT-ChR2, *n* = 8), M1 (*n* = 7), and S2 (*n* = 7), consistent with neuronal projections (*SI Appendix*, Fig. S4*B*). (*D–F*) Connectivity matrices of the somatosensory network measured by rs-fMRI versus fMRI with cortical inactivation versus neuronal tracing. For the relative connectivity strengths in somatosensory-related ROIs in each hemisphere, degrees of resting-state connectivity were normalized to the strongest connectivity with seed areas (*D*), fMRI signal changes were normalized to those in the optogenetic source region (*E*), and anatomic projection densities were normalized to those in injection site (*F*) (obtained from the Allen Institute) (19). Rs-fMRI based on temporal correlation mainly reflected bilateral homotopic connectivity, whereas spontaneous connectivity measured by fMRI with cortical inactivation and anatomical connectivity were mainly observed in ipsilaterally networked cortical and subcortical regions, indicating that the bilateral homotopic connectivity of rs-fMRI was unlikely to be due to direct CC neural communication. Red-to-orange color scale, relative connectivity strength with the seed or source areas.

First, we investigated resting-state somatosensory networks by fMRI (Fig. 2). Pertinently, rs-fMRI connectivity, which is commonly used, is determined by synchronization of fluctuating fMRI signals between regions in the absence of a task, which is presumably related to the intrinsic network of spontaneous activity. Alternatively, optogenetic cortical silencing (in a block-design paradigm with and without 20-s optogenetic stimulation; Fig. 1*A* Opto paradigm) suppresses spontaneous output activity from the stimulated site and causally reduces input to downstream networked areas (refer to actual time courses in *SI Appendix*, Fig. S6 *B–D*). Thus, the decrease in fMRI responses due to cortical silencing is closely related to the strength of resting-state connectivity between the optogenetic stimulation site and the connected regions.

Rs-fMRI data for 10-min scans (i.e., 300 volumes) without stimulation were obtained from five naïve mice; rs-fMRI connectivities in the S1FL, M1, and S2 seed regions of interest (ROIs) in the right hemisphere were mapped (Fig. 2*B*). Commonly observed strong bilateral homotopic cortical connectivity was detected without a significant network between the seed ROIs and the ipsilateral thalamus (Fig. 2*B*). Similar homotopic cortical connectivity was observed in transgenic VGAT-ChR2 mice (*SI Appendix*, Fig. S4*A*), indicating that bilateral homotopic correlation is a common feature in rs-fMRI scans. This bilateral cortical connectivity may have been due to direct CC communications and/or synchronized common neural and vascular sources. Therefore, fMRI with cortical inactivation can be used to examine the contribution of spontaneous neuronal CC communications to bilateral rs-fMRI connectivity.

Focal silencing of S1FL, M1, or S2 activity by 20 s of optogenetic stimulation of inhibitory neurons reduced CBV in networked cortical and subcortical sites (Fig. 2*C* and *SI Appendix*, Fig. S6). Inactivation of the S1FL induced negative CBV changes at the ipsilateral S1 (hindlimb and whisker barrel), M1, S2, thalamic nuclei, including the ventral posterolateral nucleus (VPL) and POm, and striatum (refer to Fig. 2*C* for group-averaged fMRI maps and *SI Appendix*, Fig. S6*B* for responses of individual animals; VGAT-ChR2, *n* = 8 mice). Similar observations were detected for M1 inactivation (refer to Fig. 2*C* for group-averaged fMRI maps and *SI Appendix*, Fig. S6*C* for responses of individual animals; *n* = 7 mice) and for the inhibition of S2 activity (refer to Fig. 2*C* for group-averaged fMRI maps and *SI Appendix*, Fig. S6*D* for responses of individual animals; *n* = 7 mice). Interestingly, the S1FL and M1 had reciprocal connections with similar magnitudes, while the CT connection was stronger in the POm than in the VPL (Fig. 2*C*). These fMRI networks are topologically consistent with anatomical neural-tracing studies (*SI Appendix*, Fig. S4*B*) (19).

To examine which resting-state somatosensory networks by rs-fMRI (measured by correlation analysis) and cortical silencing fMRI (measured by the signal reduction due to cortical inhibition in a resting state) (*SI Appendix*, Table S1) closely reflect intrinsic neural networks (measured by viral tracer injections), the relative connectivity strengths were quantified by normalization with the strongest connectivity in rs-fMRI (Fig. 2*D*), with the response at the stimulation site during cortical silencing fMRI (Fig. 2*E*) and with the projection density in tracer injection site (Fig. 2*F*). The commonly observed bilateral homotopic connections of rs-fMRI (38, 39) were weakly observed in networks during cortical inhibition fMRI and neural-tracing studies. In the degree of correlation with connectivity strength of neural-tracing data, rs-fMRI connectivity showed a poor similarity (r = 0.09), whereas networks by cortical silencing fMRI were well matched (r = 0.71). These indicate that the dominant bilateral cortical connectivity detected by rs-fMRI was unlikely due to direct neuron-based CC communication. Overall, brain-wide spontaneous networks in the resting state can be mapped by fMRI with inhibition at focal sites.

### Dissection of Somatosensory Activity-Driven Thalamic Responses by Cortical Silencing.

Somatosensory stimulation of the FP (0.5 mA, 0.5-ms duration, and 4 Hz) induced robust CBV increases in the contralateral somatosensory network (refer to *SI Appendix*, Fig. S5 for group-averaged fMRI maps and *SI Appendix*, Fig. S6*A* for responses of individual animals; *n* = 22 mice). Brain-wide activation sites were observed in the cortex, including in the S1FL, M1, S2, and thalamic nuclei, including VPL and POm (*SI Appendix*, Fig. S5*B*). This result suggests that evoked responses to FP stimulation possibly contain ST, TC, CC, and CT pathways. To dissect the functional circuits that evoked an fMRI response, optogenetic cortical inhibition was performed at the S1FL, M1, and S2 in conjunction with FP stimulation.

**Fig. 3.**
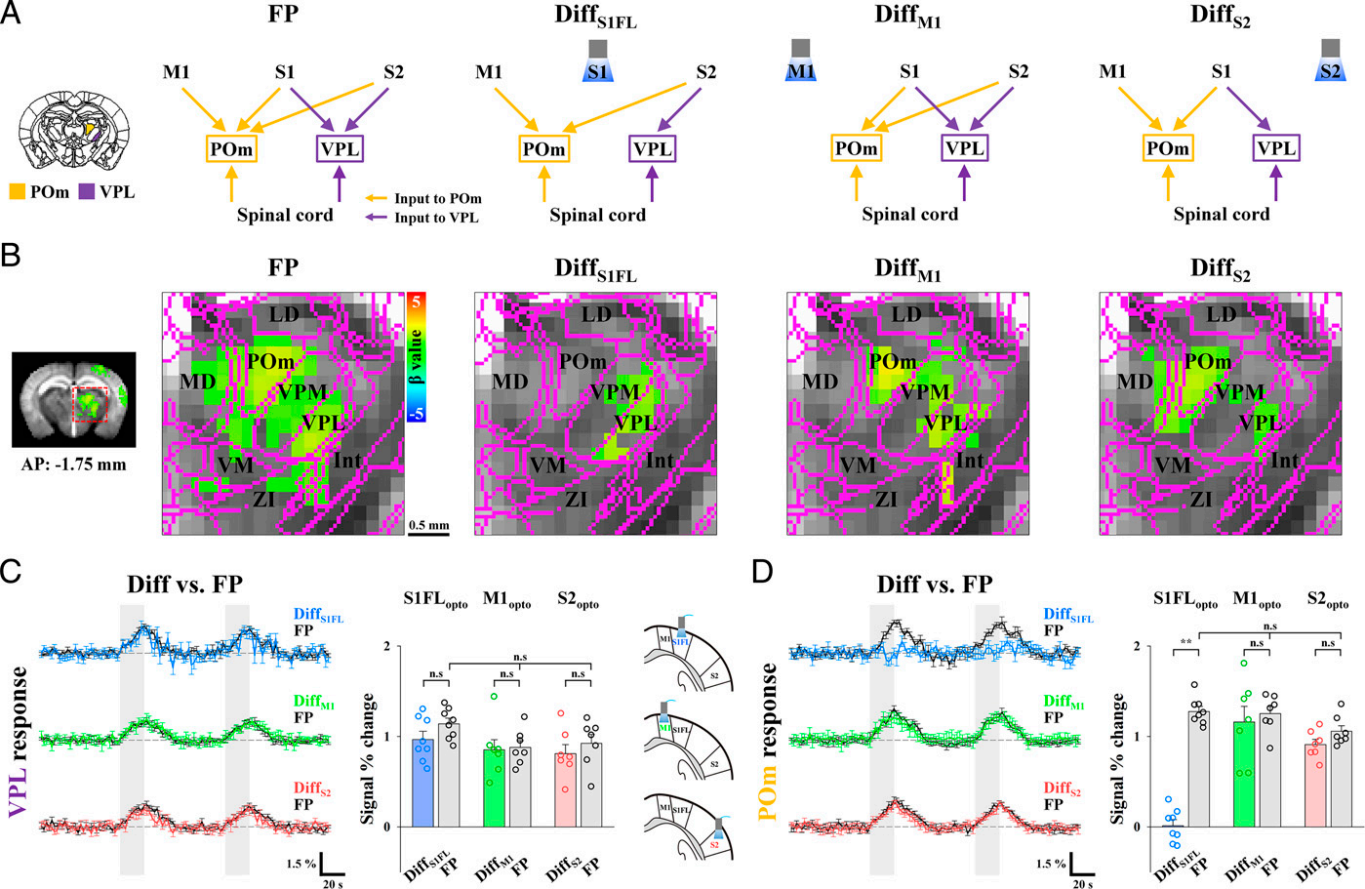
Dissection of somatosensory-driven thalamic fMRI responses with cortical silencing. (*A*) Schematic diagrams to determine the contribution of long-range inputs to somatosensory-evoked thalamic fMRI responses by means of optogenetic cortical inhibition. Functional activities in thalamic nuclei during FP stimulation can originate from multiple long-range inputs via ST and CT pathways, whereas differences in thalamic fMRI responses (Diff with a subscript indicating the inactivation site) between optogenetic cortical inactivation with and without sensory stimulation are related to all possible inputs except those from the target cortex of the S1FL, M1, and S2 to the thalamic nuclei; thus, with comparison of those conditions (Diff versus FP), the contribution of each pathway to thalamic responses was investigated. (*B*) fMRI maps of the thalamic nuclei. FP stimulation induced activity in two distinct foci, VPL and POm (FP: *n* = 22). Somatosensory-evoked activities in the VPL were maintained in the fMRI difference maps (Diff) between optogenetic cortical inactivation with and without FP, whereas POm activity disappeared in the difference map of S1FL inactivation (Diff_S1FL_: *n* = 8) but not M1 (Diff_M1_) and S2 (Diff_S2_) inactivation (*n* = 7, each). LD, lateral dorsal nucleus; MD, mediodorsal nucleus; VM, ventral medial nucleus; Int, internal capsule; and ZI, zona incerta. (Scale bar, 0.5 mm.) (*C* and *D*) Contribution of functional pathways to somatosensory-evoked VPL and POm responses. fMRI time courses in (*C*) the VPL and (*D*) the POm for the difference between combined stimulation and cortical silencing (Diff_S1FL_, blue lines; Diff_M1_, green lines; and Diff_S2_, red lines) and those for FP only (FP, black lines) were compared. (*C*) Under cortical silencing with and without FP, those differential responses (colored bar) are similar to the total VPL responses (gray bar), (*D*) whereas the sensory-evoked POm response was completely suppressed during S1FL silencing (blue bar) but not during M1 (green bar) and S2 (red bar) silencing. Therefore, the VPL has dominant inputs from the spinal cord, not the cortex, and POm activity is directly driven by the S1FL, not by the spinal cord. Gray vertical bar in time courses, 20-s stimulus; error bars, SEM; n.s., not significant; and **P < 0.01 (paired *t* test).

Initially, we investigated the relative contribution of ST and CT circuits to thalamic fMRI responses (Fig. 3). To separate these two contributions to thalamic fMRI signals, fMRI responses to optogenetic silencing of the targeted cortex (refer to individual animal data in *SI Appendix*, Fig. S6) were compared to those of simultaneous silencing and FP stimulation (refer to individual animal data in *SI Appendix*, Fig. S7). These differences are directly related to all inputs except the CT inputs from the target site to the thalamic nuclei (Diff_S1FL_, Diff_M1_, and Diff_S2_ in Fig. 3*A*). In the VPL, the CBV response to somatosensory stimulation was similar to the difference between cortical silencing outcomes and silencing/FP stimulation outcomes (Diff), regardless of S1FL, M1, or S2 silencing (Fig. 3 *B* and *C*); this finding suggests that the VPL is driven by inputs from the spinal cord, not from the cortex. In the POm, S1FL silencing suppressed fMRI responses to FP stimulation completely, whereas M1 and S2 silencing had no impact (Fig. 3 *B* and *D*), indicating that POm activity was directly driven by S1, not by ST inputs. With our fMRI approach, the relative contributions of feedback CT and feedforward ST activity to sensory-evoked thalamic responses were successfully determined.

### Dissection of Somatosensory-Evoked S1FL and M1 Responses to TC, CC, and IC Activity.

**Fig. 4.**
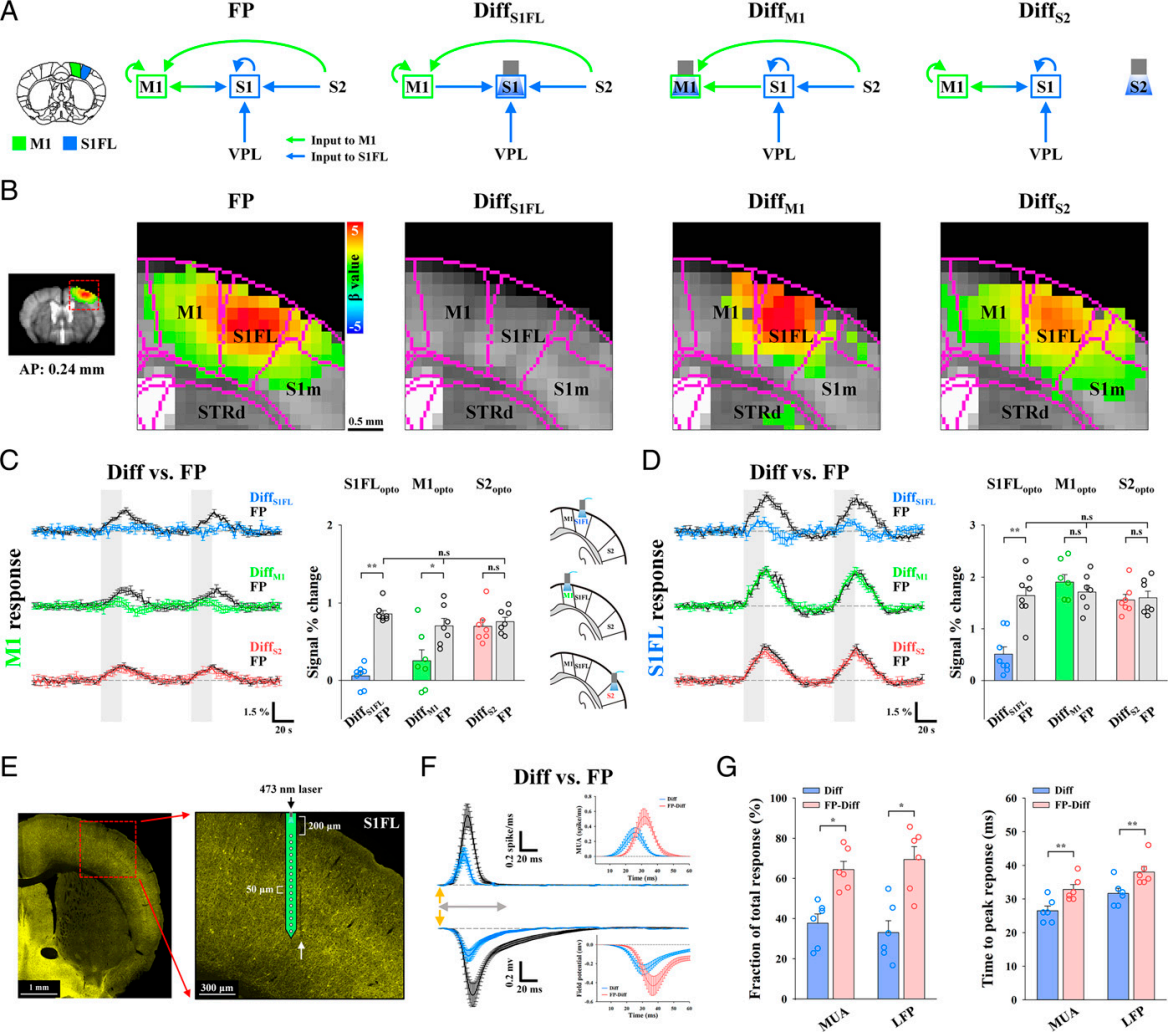
Dissection of TC, CC, and IC activity during somatosensory fMRI responses in the S1FL and M1. (*A*) Schematic diagrams to determine the contribution of long-range and local activity to somatosensory-evoked M1 and S1FL responses with optogenetic cortical inhibition. Functional activity in the S1FL during FP stimulation can originate from both long-range inputs via TC and CC pathways and local IC activity, whereas M1 responses can comprise CC inputs and IC activity. The differences in cortical responses between optogenetic cortical inactivation with and without sensory stimulation (Diff) are related to input-driven activity, excluding CC and/or IC activity from the inactivation site (indicated as a subscript). (*B*) fMRI maps in cortical areas including M1 and the S1FL. Somatosensory-evoked activities in M1 (FP: *n* = 22) disappeared in the fMRI difference map between M1 (Diff_M1_: *n* = 7) or the S1FL (Diff_S1FL_: *n* = 8) optogenetic inactivation with and without FP stimulation but remained after S2 inactivation (Diff_S2_: *n* = 7), whereas somatosensory-evoked S1FL responses disappeared only after S1FL inactivation. S1m, primary somatosensory area of mouth and STRd, striatum of dorsal region. (*C* and *D*) Contribution of functional pathways to somatosensory-evoked M1 and S1FL responses. fMRI time courses in (*C*) M1 and (*D*) S1FL to determine the difference between combined stimulation and cortical silencing (Diff_S1FL_, blue lines; Diff_M1_, green lines; and Diff_S2_, red lines) and those for FP stimulation (FP, black lines) only were compared. (*C*) The differential response in M1 (green bar) between M1 silencing with and without FP stimulation was approximately one-third of the total M1 response (gray bar). In addition, the sensory-evoked M1 response was mostly suppressed after S1FL silencing (blue bar) but not after S2 silencing (red bar). (*D*) The difference in S1FL responses between S1FL silencing with and without FP stimulation (blue bar) is related to TC input, which accounts for approximately one-third of the total S1FL response (gray bar). However, sensory-evoked S1FL responses were not changed by M1 (green bar) and S2 (red bar) silencing. Therefore, M1 fMRI responses were dominantly driven by the S1FL, and S1FL fMRI responses were directly driven by the VPL, with minimal CC contribution; both of these responses were amplified by IC circuits. Gray vertical bar in time courses, 20-s stimulus; error bars, SEM; n.s., not significant; and *P < 0.05 and **P < 0.01 (paired *t* test). (*E–G*) Electrophysiological recording at 200- to 1,000-µm depth with the 16-channel opto-electrode performed to investigate the neural source of the S1FL fMRI response. (*E*) Fluorescence microscopy image from a representative animal shows the expression of ChR2-EYFP (yellow) in the S1FL and the location of the electrode track (white arrow). (*F*) Neural data were time-lock averaged for the FP stimulus interval of 250 ms. Depth-averaged neural responses (MUA and LFP, VGAT-ChR2, *n* = 6) in the S1FL for the difference between combined stimulation and S1FL silencing (Diff, blue lines) and those for FP stimulation (FP, black lines) only were compared to isolate the local IC activity (FP-Diff, red lines in *Inset*). Detailed depth-dependent responses are shown in *SI Appendix*, Fig. S8. (*G*) Fraction and time to peak of sensory inputs (blue bar) and IC activity (red bar) in MUA and LFP. Quantitative MUA and LFP values are reported in *SI Appendix*, Tables S2 and S3. Yellow arrows in *F*, 0.5-ms FP stimulus; gray double arrows in *F*, 60-ms period for the *Inset* figure; error bars, SEM; n.s., not significant; and *P < 0.05 and **P < 0.01 (paired *t* test).

Next, we examined the relative contribution of long-range inputs and local circuits to fMRI responses in the S1FL and M1 (Fig. 4). Somatosensory-induced fMRI responses of the S1FL can contain TC input from the VPL, IC activity, and CC feedback from M1 and S2, whereas fMRI responses of M1 can contain CC inputs and IC activity. The role of each pathway in somatosensory-evoked fMRI responses in S1FL and M1 was examined by optogenetic silencing of the S1FL, M1, or S2 (refer to Fig. 4*A* for input circuit diagrams and Fig. 4*B* for corresponding fMRI maps; refer to individual animal traces in *SI Appendix*, Figs. S6 and S7).

We first examined the contributions of functional pathways to somatosensory-evoked M1 responses (Fig. 4*C*). M1 responses were completely suppressed when optogenetic S1FL silencing was performed (Diff [blue] versus FP [black/gray] in Fig. 4*C*) but remained the same when S2 activity was silenced (Diff [red] versus FP [black/gray] in Fig. 4*C*). This suggests that somatosensory-evoked M1 activity is mostly driven by CC projections from the S1FL. CC input-driven magnitude responses during M1 silencing accounted for ∼36% of the total somatosensory-evoked fMRI response in M1 (Diff [green bar] versus FP [gray bar] in Fig. 4*C*). Since the input-driven CBV response duration (green line) was shorter than the duration of the total CBV response (black line), the areas under the curve (AUCs) for these measurements were also compared; this analysis revealed that the input-driven CBV response accounted for 24% of the total response. M1 somatosensory-evoked activity was shown to be driven by the S1FL feedforward projection and amplified via the IC circuitry.

Then, the contributions of the TC, IC, and CC circuits to S1FL responses were separated (Fig. 4*D*). Based on fMRI data acquired after M1 or S2 silencing (green or red), we found that CC projections from M1 and S2 to the S1FL were negligible during FP stimulation. This result indicated that there was no feedback CC long-range contribution to the sensory response in the S1FL, as expected under anesthesia (40, 41). When IC activity in the S1FL was suppressed, a positive CBV response remained, with a reduced magnitude of ∼31% of that of the total stimulus-driven response (Diff [blue] versus FP [black/gray] in Fig. 4*D*) and 26% of that of the total AUC.

To investigate the neural source of the somatosensory-induced fMRI reduction caused by local silencing, 16-channel electrophysiological recordings with the same stimulus paradigms as fMRI (Fig. 4*E*; VGAT-ChR2, *n* = 6 mice) were acquired in the S1FL. Time-locked neural data for one FP stimulus-pulse interval of 250 ms were obtained by averaging all 250-ms windows (FP; black in Fig. 4*F*). Inhibitory neuronal activities induced by optogenetic stimulation of GABAergic neurons were subtracted from the difference between those responding to optogenetic stimulation with and without somatosensory stimulation (Diff; blue). MUAs and local field potentials (LFPs) were compared across different experimental conditions (refer to Fig. 4 *F* and *G* for averaged responses across all 16 channels and *SI Appendix*, Fig. S8 for depth-dependent responses). The difference between optogenetic silencing of S1FL and simultaneous silencing/FP stimulation (Diff; blue) indicated neural activities induced by TC input, whereas somatosensory-driven activity (FP) contained both long-range input and local recurrent activity (Fig. 4*F*). Thus, the local recurrent activity (FP-Diff; red) can be obtained from the difference between long-range TC input (Diff; blue) and total somatosensory activity induced by forepaw stimulation (FP; reference inset figures in Fig. 4*F*). The thalamic input activity integrated over time was ∼35% of the forepaw-induced neural activity (Fig. 4*G*; 37.73 ± 4.54% for MUA and 33.03 ± 5.92% for LFPs; refer to *SI Appendix*, Table S2 for MUA and *SI Appendix*, Table S3 for LFP), which is in good agreement with the fMRI observations (31% for magnitude and 26% for AUC). The somatosensory-evoked local recurrent activity suppressed by optogenetic silencing occurred ∼6 ms later than the input activity (Fig. 4*G*; Diff versus FP-Diff; 26.50 ± 1.43 ms versus 32.83 ± 1.45 ms for MUA; 31.67 ± 1.52 ms versus 38.00 ± 1.71 ms for LFPs; refer to *SI Appendix*, Table S2 for MUA and *SI Appendix*, Table S3 for LFPs). Combined with macroscopic fMRI and microscopic electrophysiology data, these findings showed that somatosensory-evoked TC activity was amplified approximately twofold in the S1FL due to local recurrent circuits (15) and demonstrated that fMRI can be used to separate long-range input and local circuit contributions.

**Fig. 5.**
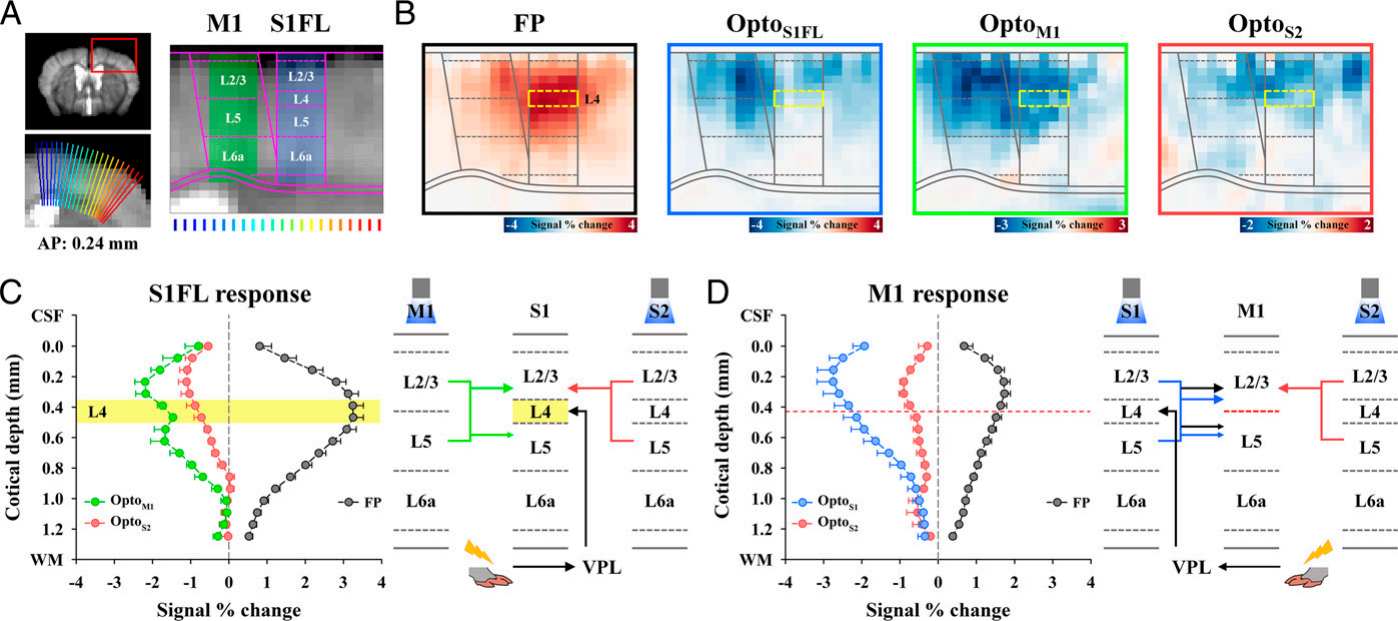
The highest CBV responses at synaptic input layers in the S1FL and M1. (*A*) Cortical flattening for layer-specific fMRI analysis in the S1FL and M1. The cortical area (red box) was upsampled to double using bicubic interpolation and linearized using radially projecting lines (blue-to-red) perpendicular to the cortical edges (underlay, study-specific brain template; overlay, Allen Mouse Brain Atlas). Laminar boundaries for each cortex were defined as the laminar thickness distribution. (*B*) Cortical depth-dependent fMRI maps with nominal 78-µm in-plane resolution for somatosensory-evoked and spontaneous activities. Somatosensory-evoked activities (FP: *n* = 22) were localized at L4 of the S1FL (yellow-dashed box) and L2/3 of M1. Strong suppression occurred at L2/3 of the S1FL and M1 during optogenetic cortical inactivation (Opto_S1FL_: *n* = 8, Opto_M1_: *n* = 7, and Opto_S2_: *n* = 8). (*C* and *D*) Cortical depth-dependent CBV profiles and expected circuit diagrams. Signal changes were averaged for the same depth and plotted as a function of distance from the surface in (*C*) the S1FL and (*D*) M1. (*C*) The somatosensory-evoked response (black) peaked at L4 of the S1FL, while M1 inactivation (green) induced two cortical peaks at L2/3 and L5; in addition, S2 silencing (red) induced the largest changes at L2/3. Since the ∼150-µm-thick L4 of the S1FL does not receive inputs from M1, the cortical profile responding to M1 stimulation indicates that the PSF of laminar CBV response is less than 150 µm FWHM. (*D*) The largest fMRI changes in M1 were mostly detected at L2/3, regardless of the type of stimulus used. Individual animal profiles are shown in *SI Appendix*, Fig. S9.

Then, we examined whether TC and CC input layers in the S1FL and M1 can be identified by upsampled cortical depth-dependent CBV responses (Fig. 5). Somatosensory-evoked CBV responses peaked at layer 4 (L4) of the S1FL (refer to Fig. 5 *B* and *C* for group-averaged fMRI responses and *SI Appendix*, Fig. S9*A*, *i* for responses of individual animals; *n* = 22 mice), indicating the TC input, and peaked at layer 2/3 (L2/3) of M1 (refer to Fig. 5 *B* and *D* for group-averaged fMRI responses and *SI Appendix*, Fig. S9 for responses of individual animals; *n* = 22 mice), indicating the CC input. Cortical suppression in the S1FL, M1, and S2 induced negative CBV changes in the optogenetic stimulation site and peak responses in CC input layers of networked regions (refer to Fig. 5 *B–D* for group-averaged fMRI responses and *SI Appendix*, Fig. S9 for responses of individual animals). When M1 was suppressed, the CC projection occurred at L2/3 and L5A, not L4, in S1 (42). Corresponding CBV peaks with different magnitudes were detected, with a small dip at ∼150-µm-thick L4 (Fig. 5*C*, green), indicating that CBV responses exhibit a hemodynamic point spread function (PSF) of <150 µm full width at half maximum (FWHM). Our laminar-resolved fMRI data showed that cortical CBV responses peaked at synaptic input layers with high specificity.

### Separation of the TC, CC, and Cortico-Thalamo-Cortical Contributions to Somatosensory-Evoked S2 Responses.

**Fig. 6.**
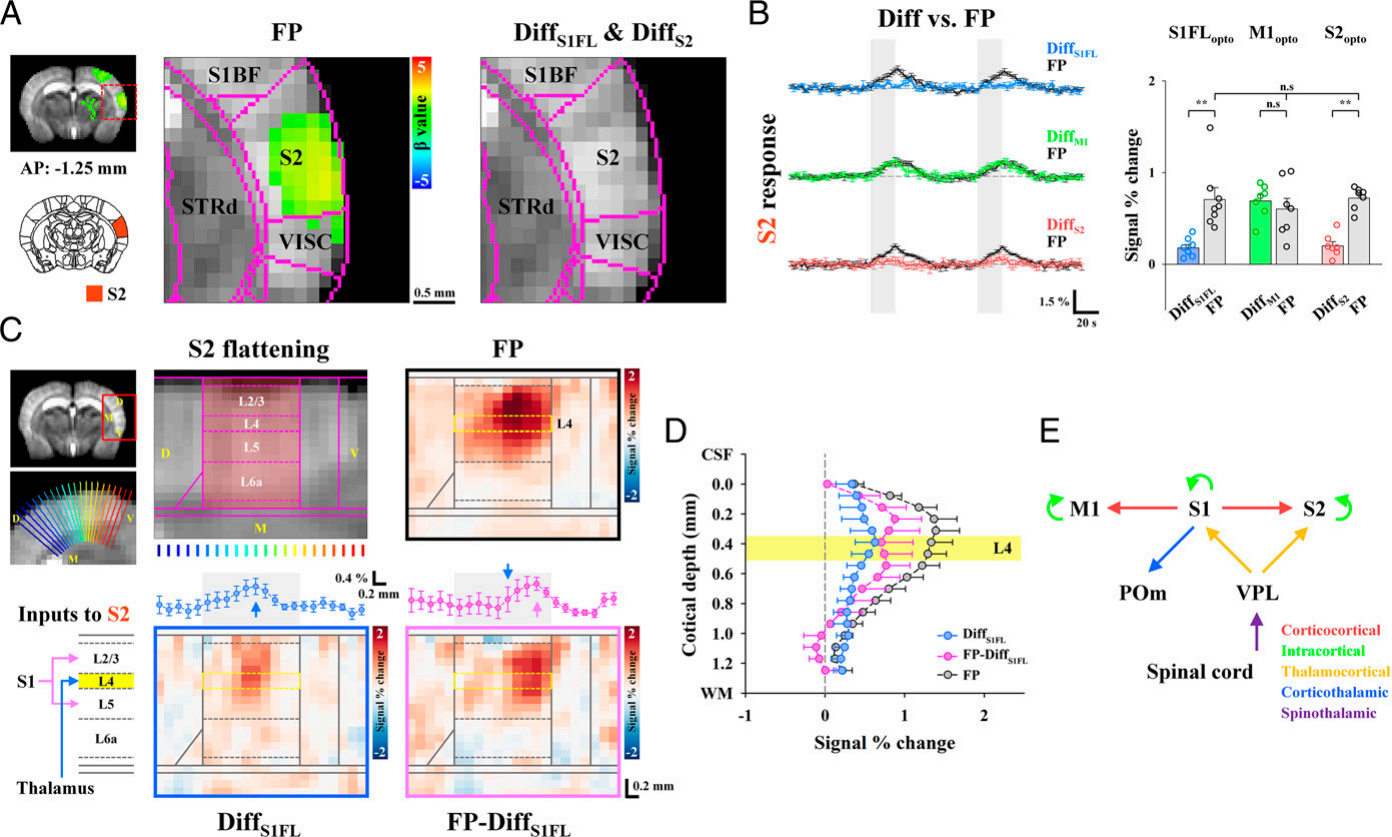
Separation of TC, CC, and CTC contributions to sensory-evoked fMRI responses in S2. (*A*) fMRI maps in cortical areas including S2. Somatosensory-evoked activities in S2 (FP: *n* = 22) disappeared in the fMRI difference map between S1FL or S2 optogenetic inactivation with and without FP stimulation (Diff_S1FL_: *n* = 8 and Diff_S2_: *n* = 7) but remained after M1 inactivation. S1BF, primary somatosensory area of the barrel field; VISC, visceral area; and STRd, striatum of dorsal region. (*B*) Contribution of functional pathways to the somatosensory-evoked S2 response. fMRI time courses in S2 for the difference between combined stimulation and cortical silencing (Diff_S1FL_, blue line; Diff_M1_, green line; and Diff_S2_, red line) and those obtained for FP stimulation only (FP, black lines) were compared. Under S2 silencing with and without FP stimulation, the difference response in S2 (red bar) was approximately one-third of the total S2 response (gray bar). The sensory-evoked S2 response was largely suppressed in S1FL silencing (blue bar) but still had TC input (∼25% of the total S2 response) and was not changed by M1 silencing (green bar). Therefore, the S2 fMRI response was predominantly driven by the S1FL with minor VPL contribution and was amplified by IC circuits. Gray vertical bar in time courses, 20-s stimulus; error bars, SEM; n.s., not significant; and **P < 0.01 (paired *t* test). (*C*) Layer-specific fMRI analysis in S2. Thalamic inputs, including both TC and CTC circuits, project to L4, whereas direct S1FL inputs project to L2/3 and L5 (Inputs to S2). To investigate the laminar origin of the evoked S2 response, the S2 area was upsampled and linearized using radially projecting lines (blue-to-red) perpendicular to the cortical edges (underlay, study-specific brain template and overlay, Allen Mouse Brain Atlas). Laminar boundaries were defined as cortical thickness distribution (S2 flattening). To separate layer-specific TC inputs and direct/indirect S1FL inputs to S2 from the total sensory-evoked response, fMRI responses for the difference between combined stimulation and S1FL silencing (Diff_S1FL_, TC inputs) and those for FP stimulation only (FP from only dataset paired with Opto_S1FL_) were compared (FP-Diff_S1FL_, direct/indirect S1FL inputs). In S2 flattened maps, somatosensory-evoked responses were averaged across the cortical layers and plotted in a dorsal-to-ventral direction (left-to-right). The patches responding to TC (blue profile in Diff_S1FL_) and direct/indirect S1FL (pink profile in FP-Diff_S1FL_) inputs were separable. Blue (Diff_S1FL_) and pink (FP-Diff_S1FL_) arrows, locations of the response peak; and error bars, SEM. (*D*) For the cortical depth-dependent profile, signal changes were averaged for the same depth and plotted as a function of distance from the surface in S2. During the sensory-evoked response in S2 (FP, black line), the laminar profile for TC inputs peaked at L4 of S2 (Diff_S1FL_, blue line), while the cortical profile projected from S1FL was observed to have double-peak responses at L2/3 and L5 (FP-Diff_S1FL_, pink line), indicating direct CC inputs. Yellow region, layer 4 and error bars, SEM. (*E*) Putative functional circuits in the somatosensory network as measured by CBV-weighted fMRI with optogenetic cortical silencing.

Finally, we examined the relative contribution of long-range TC, CC and cortico-thalamo-cortical (CTC) inputs and local circuits to fMRI responses in S2 (Fig. 6). The S2 area is anatomically connected to the VPL, POm, S1FL, and M1 (43). Thus, functional inputs to S2 during FP stimulation can originate from monosynaptic circuits from the VPL (TC) and S1FL (CC) and disynaptic projections from the S1FL via M1 (S1FL → M1 → S2) and the POm (S1FL → POm → S2). When M1 activity was suppressed, fMRI responses at S2 remained the same (Diff [green] versus FP [black/gray] in Fig. 6*B*), indicating that the contribution of the S1 → M1 → S2 pathway was negligible. The contribution of the TC projection, calculated by the difference between the signal change without and with S1FL silencing (refer to *SI Appendix*, Figs. S6 and S7 for individual animal traces), constituted 25% of the total S2 response (Diff [blue] versus FP [black/gray] in Fig. 6*B*), whereas the remaining 75% of inputs originated from the S1FL. When the sensory-evoked S2 fMRI responses were compared without and with S2 silencing, CBV responses of long-range synaptic inputs accounted for ∼28% of the total percent change in S2 (Diff [red] versus FP [black/gray] in Fig. 6*B*; 30% of the total AUC), whereas the remaining responses were due to local circuit contributions. These findings suggested that somatosensory-evoked S2 activity was mostly driven by feedforward S1FL inputs with minor VPL inputs and amplified by the IC recurrent circuit.

The synaptic inputs from the S1FL to S2 can be direct S1FL (CC) inputs and/or indirect S1FL (CTC) inputs via POm (44). Thus, our next question was whether the direct S1FL input to S2 (S1FL → S2) can be separated from CTC input (S1FL → POm → S2). Since direct S1FL inputs project to layers 2/3 (L2/3) and L5 and CTC inputs project to L4 (layer “Inputs-to-S2” schematic in Fig. 6*C*) (42, 44, 45), upsampled cortical, depth-dependent analysis can provide an indication of whether CTC inputs are dominant in S2 fMRI responses (Fig. 6*C*). Since laminar CBV responses are specific to synaptic input layers with a PSF of <150 µm, its peak position provides information on cortical input layers. During FP somatosensory stimulation, laminar S2 responses were broad around L2/3 and L4 (FP in Fig. 6*C* and the black profile in Fig. 6*D*). To obtain somatosensory-evoked fMRI signals originating from the VPL, the difference in cortical responses between S1FL inactivation with and without FP stimulation was obtained (Diff_S1FL_ in Fig. 6*C* and blue profile in Fig. 6*D*). Then, the S1FL input (FP-Diff_S1FL_ in Fig. 6*C* and the pink profile in Fig. 6*D*) to S2 was determined by subtracting the VPL input (Diff_S1FL_) from the total sensory-evoked response (FP). The TC inputs peaked at L4 in the medial S2 (Diff_S1FL_ in Fig. 6*C* and blue profile in Fig. 6*C*), whereas the inputs from the S1FL induced double peaks at L2/3 and L5 in the ventral S2 (FP-Diff_S1FL_ in Fig. 6*C* and the pink profile in Fig. 6*D*), indicating direct CC projections. These TC and CC inputs to S2 are spatially segregated by ∼0.47 mm in a dorsal–ventral direction (Diff_S1FL_ [blue arrow] versus FP-Diff_S1FL_ [pink arrow] in Fig. 6*C*), which cannot be easily identified by microscopic tools with a small field of view. Based on our laminar-resolved CBV fMRI with cortical silencing, we successfully confirmed TC and direct CC inputs to S2.

Overall, we completely scrutinized the relative contributions of somatosensory-driven, long-range, and local recurrent circuits to fMRI responses with the aid of focal optogenetic silencing and laminar-specific CBV contrasts (Fig. 6*E*).

## Discussion

We developed fMRI approaches with highly specific CBV contrasts and local optogenetic silencing to dissect resting-state and functional brain-wide, long-range networks. Pertinently, we successfully determined the relative contribution of each somatosensory circuit to fMRI responses in mice at the population level. VPL responses originated mostly from the spinal cord, whereas the POm received functional CT projections from the S1FL (46, 47). The S1FL received somatosensory input from the VPL (44, 48), and M1 received feedforward CC input from the S1FL. S2 received mostly direct CC input (∼75%) from the S1FL and a small amount of TC input (∼25%) from the VPL. The long-range synaptic input in cortical areas was amplified approximately twofold by local IC circuits. Since our findings are consistent with electrophysiology studies at preselected sites (13–16, 22, 24, 44, 46), the fMRI approach to long-range circuit analysis is viable for mapping long-range functional circuits in the whole brain.

The fMRI approach with cortical silencing can be extended for investigations of brain-wide functional circuits employing external stimuli or direct cortical stimulation. Evoked stimulation can be achieved via external or IC approaches with optogenetic or electric stimulation, activating entire networks without knowledge of the exact flow direction except for the target site. Thus, localized silencing is necessary to suppress downstream networks; this suppression can be achieved by optogenetic stimulation with high temporal specificity and pharmacological and photochemical tools for bulk interventions for specific neurotransmitter inputs to a defined brain region with low temporal specificity (10). The advantage of optogenetic tools is that they provide users with the ability to perform fMRI experiments under external stimulation with and without cortical silencing in an interleaved manner; this ability is essential for signal averaging without bias by slowly modulating animal physiology during imaging studies.

### Focal Inhibition by Optogenetic Stimulation of Inhibitory Neurons.

Optogenetic activation of cortical GABAergic neurons has been widely used in circuit/systems neuroscience (15, 35). To identify the contribution of neural circuits to fMRI responses with optogenetic stimulation of the selected region in VGAT-ChR2 mice, it was assumed that cortical GABAergic interneurons were mostly local. Based on Allen mouse brain connectivity (https://connectivity.brain-map.org/projection/experiment/167441329), long-range projections of GABAergic interneurons were found to be negligible in the S1 barrel field. Our assumption appears to be valid in the somatosensory cortex.

Another assumption was that optogenetic stimulation of GABAergic interneurons fully suppresses excitatory recurrent neuronal activities across cortical depths and downstream projections. Although the penetration depth of blue light is dependent on laser power, it is quite shallow (depth of half maximum = ∼300 µm) (34). In our MUA measurements in the S1FL, optogenetic stimulation of inhibitory neurons reduced spontaneous MUA by ∼90% across cortical layers (Fig. 1*B* and *SI Appendix*, Fig. S2*B*) and suppressed somatosensory stimulation-induced IC recurrent circuits (Fig. 4*F*); these outcomes are possibly due to local circuits within a cortical column. In our fMRI studies, the somatosensory-evoked responses in the POm and M1 were completely suppressed when inhibitory neurons in the upstream S1FL were optogenetically stimulated, suggesting that cortical inhibition is effective in blocking downstream projections.

The spatial extent of cortical inhibition is important to determine the partial volume of inhibition within the photostimulated cortical region (S1FL, M1, and S2). Li et al. (34) measured the spatial spread of inhibition beyond the target area of the somatosensory cortex in VGAT-ChR2 mice. Neural activity is suppressed by >70% for 1.5-mW photostimulation and >90% for 14 mW at 1 mm away from the stimulation site, far beyond the spatial spread of light (0.25 to 0.5 mm). In our case, with 3 mW optogenetic stimulation of VGAT-ChR2, the spatial spread of inhibition was expected to be >1 mm from the center of the fiber. Since the sizes of S1FL, M1, and S2 in mice are ∼0.84 × 1.69, 0.82 × 1.47, and 1.56 × 1.84 mm^2^, respectively, based on the Allen Mouse Brain Atlas, the ROI was less than the area inhibited by optogenetic stimulation of GABAergic neurons. Therefore, our assumption of full inhibition is valid.

### Hemodynamic Responses to Inhibitory Neuronal Activity.

Hemodynamic fMRI responses are believed to be mostly driven by excitatory activity (8). Although GABAergic interneuron activity is known to regulate local vascular tone by the release of vasoactive mediators (e.g., nitric oxide) (49), their activity also interacts with nearby excitatory neurons in the cortex. When inhibitory neurons were activated in VGAT-ChR2 mice, an increase in CBF and CBV was observed for <5 s of stimulation (37, 50, 51), indicating that inhibitory neurons indeed increase hemodynamic responses. However, the increased inhibitory activity by optogenetic stimulation suppressed excitatory activity (inhibition), which led to a decrease in hemodynamic responses. Therefore, hemodynamic responses may be closely dependent on stimulus frequency and duration. Notably, we recently investigated the contribution of inhibitory neuronal activity to fMRI responses using multimodal measurements with electrophysiology, BOLD fMRI, and optical-imaging during ChR2 stimulation of inhibitory neurons in VGAT-ChR2 mice (37). Under the same stimulation parameters, biphasic BOLD fMRI and CBV-weighted optical-imaging responses were observed at the stimulated site with increased inhibitory and decreased excitatory neuronal activity; an initial small positive change (by increased inhibitory activity) was followed by a prolonged negative response (by suppressed excitatory activity). In our CBV fMRI data, a biphasic response in the S1FL during the stimulation period and poststimulus overshoot were observed. The initial CBV increase was directly due to inhibitory neuron activity, as often seen in hemodynamic studies with short stimulation, and the prolonged negative change was due to the suppression of excitatory neurons (inhibition). A balance between excitation of inhibitory neurons and inhibition of excitatory neurons changes the magnitude and polarity of hemodynamic responses.

### Laminar PSF of CBV Responses.

The resolving power of laminar fMRI across different layers is closely dependent on fundamental CBV point spread and voxel resolution. Spatial specificity to neuronally active layers is improved with stimulation time up to ∼10 s (52, 53), which may occur due to different dynamics of macro- and microvessels, fast-acting penetrating arterioles, and highly specific slow-responding capillaries (54). The CBV PSF was previously measured in the S1FL of mice with CBV-weighted optical-imaging (55) and in the rat olfactory bulb with CBV-weighted fMRI (56); the PSF was found to be ∼100 µm FWHM. In our studies with nominal 78-µm in-plane resolution, we can resolve laminar responses within the ∼1-mm-thick somatosensory cortex. Two peaks at L2/3 and L5 in the S1FL were observed during optogenetic stimulation in M1, albeit with interanimal variation (*SI Appendix*, Fig. S9), which was expected based on a previously determined CBV PSF (55).

### Mapping Spontaneous Neural Communication.

In rodent rs-fMRI, a strong correlation occurs between bilateral homotopic cortices (38, 39) but not between the cortex and thalamus in the ipsilateral hemisphere. The strong bilateral homotopic correlation is often explained by direct CC connections (38, 57) due to the existence of monosynaptic anatomical projections (19). However, careful evaluation of anatomical tracing data show that ipsilateral projections among the somatosensory networks (including thalamus) are generally larger than contralateral homotopic projections (Fig. 2*F* and *SI Appendix*, Fig. S4*B*) (19). Notably, rs-fMRI fails to detect strong ipsilateral connectivity between the cortex and thalamic nuclei within the somatosensory network; such connectivity was indeed observed in spontaneous connectivity maps generated by focal cortical inhibition. These results suggested that conventional rs-fMRI correlation strength does not truly reflect anatomical monosynaptic connections but rather common bilateral fluctuations caused by modulatory cholinergic inputs (58–60), noradrenaline driven by the locus coeruleus (59), and thalamic low frequency (61). Further systematic studies are necessary to determine the origin of rs-fMRI connectivity.

Alternatively, brain-wide spontaneous connectivity can be determined by fMRI with optogenetic silencing. In our studies, ipsilateral connections were predominant among the somatosensory network, while the connection strength between bilateral homotopic regions was ∼30% of the ipsilateral connection strength (Fig. 2*E*). These spontaneous connectivity findings were consistent with anatomical tracing data (Fig. 2*F*) (19). Furthermore, ipsilateral, somatosensory-related regions responding to cortical inhibition overlapped with sites that were active during somatosensory stimulation. These results indicated that fMRI with cortical silencing can detect functionally networked regions. Spontaneous CT communication to the higher-order thalamic nucleus POm was stronger than that to the relay thalamic nucleus VPL, which was consistent with electrophysiological findings (12, 46).

Spontaneous downstream neural network strength can be measured by fMRI with cortical silencing. In our studies, the spontaneous network strength in S1FL → M1 and S2 (−2.75% and −1.98% of the peak response in L2/3) was larger than the somatosensory-evoked response (1.74% and 1.39% of the peak response). The relatively high spontaneous activity was surprising but may have been due to the use of ketamine for anesthesia (*Effect of Anesthesia on Resting-State and Evoked fMRI*).

The important implication of mapping spontaneous neural communication is the identification of potential circuits that modulate behavior. Optogenetic inhibition has been used to elucidate the involvement of brain regions and specific cell populations associated with behavior. However, the neural circuits involved in behaviors could act through local or downstream circuits. Although anesthesia changes the strength of long-range connections, downstream circuits associated with behavior can be mapped by fMRI.

### Long-Range Input versus Local Circuit Contributions to Sensory-Evoked fMRI.

Somatosensory-evoked long-range circuits were successfully dissected by fMRI. The CC circuit of S1 and M1 has been extensively investigated anatomically and physiologically (42, 62–64). S1 and M1 are reciprocally connected; neural excitation in S1 rapidly propagates into neurons in L2/3 and L5A in M1, which reciprocally activate neurons in L2/3 and L5A in S1 via a feedback loop (63). In our studies (Fig. 5 for the S1FL and M1 fMRI responses to FP stimulation and cortical inhibition), feedforward CC projection from S1 to L2/3 in M1 was observed for evoked and spontaneous conditions; however, reciprocal feedback projection from M1 to L2/3 and L5 in S1 was observed only for spontaneous, not evoked, conditions. It should be noted that underlying neuronal features of the downstream targets in CC projections cannot be determined with our fMRI approach, since synaptic inputs to both excitatory and inhibitory neurons can increase fMRI responses. Similarly, the sensory-evoked feedforward CC projection from S1 to L2/3 and L5 in S2 was observed without the reciprocal feedback projection. Although the S2 contributions to sensory-evoked responses in S1 and M1 were negligible in our studies, these projections may significantly contribute in other behavioral contexts, such as perception and decision-making (65–67). The differences between our study and physiological studies were likely due to anesthesia (40). To examine functional brain-wide circuits relevant to behavior, it is essential to perform fMRI on mice while they are active.

In our studies, the long-range inputs and IC circuit contributions to somatosensory-evoked fMRI responses were separated. The long-range, synaptic, input-driven fMRI response in the S1FL, S2, and M1 accounted for ∼30% of the total somatosensory-evoked response. Similarly, somatosensory-evoked MUA and LFP in the S1FL were reduced to ∼35% of the total evoked neural activity by S1FL-silencing. Therefore, the suppressed response, ∼70% of the total fMRI response (∼65% for MUA and LFP), was related to local neural activity. Our finding of a greater-than-twofold increase in the IC fMRI signal was highly consistent with electrophysiology data showing an ∼2.2-fold amplification of IC in the barrel cortex (13), a 2.4-fold amplification of IC in the primary auditory cortex (14), and a threefold amplification of IC in the primary visual cortex (15), in which TC and IC activities were separated with recordings of excitatory cells in L4 after optogenetic excitation of inhibitory neurons. Overall, fMRI with and without cortical inhibition can be used to identify the contributions of long-range input versus IC circuits, facilitating the detection of cortical excitatory/inhibitory imbalance and consequent dysfunction of IC recurrent circuits.

We investigated the contribution of long-range cortical inputs to the POm and the VPL in the thalamus, which is consistent with a previous electrophysiology study in which somatosensory-evoked responses in the higher-order POm were drastically reduced after S1 inhibition, while first-order ventral posteromedial nucleus responses were not modulated (46). According to our data, the medial thalamic area adjacent to the POm was active during somatosensory stimulation and S1FL silencing. This observation may have been due to the spillover of POm activity with limited spatial resolution and group averaging and/or due to real activation in intralaminar nuclei, which are associated with multimodal sensory activity (68). Thus, fMRI experiments with higher spatial resolution are needed to determine whether medial thalamic activity is genuine.

### Effect of Anesthesia on Resting-State and Evoked fMRI.

Anesthesia affects fMRI responses in both resting and functional states. Pertinently, ketamine is known as a dissociative anesthetic that antagonizes N-methyl-D-aspartate receptors preferentially binding to GABAergic interneurons at a low dose (69, 70). Ketamine disinhibits basal firing of excitatory pyramidal neurons, leading to an increase in electroencephalographic activity, metabolic rate, and cerebral blood flow (71). Ketamine anesthesia induces higher bilateral, homotopic, calcium-based connectivity with less spatial focality than that observed in the awake condition (72). In the functional state, ketamine (with xylazine) increases recurrent IC excitation (73) and cortical fMRI responses (74), possibly due to disinhibition compared to that demonstrated in the awake condition. Overall, it is likely that ketamine anesthesia enhances resting-state and evoked fMRI responses in cortical areas compared to the fMRI responses noted in the awake condition.

## Conclusion

Here, we successfully dissected the brain-wide somatosensory circuits underlying fMRI responses. Our fMRI approach combining stimulation and focal inhibition provides an avenue for investigating population-based neural circuits throughout the whole brain, allowing longitudinal investigations of functional reorganization caused by neuropathological modifications and learning in individual animals. Circuit-level analysis of whole-brain fMRI will complement conventional microscopic functional circuit studies by elucidating brain-wide, population-based information processing.

## Materials and Methods

All experiments were performed under ketamine and xylazine anesthesia (75) with the approval of the Institutional Animal Care and Use Committee of Sungkyunkwan University in accordance with the standards for humane animal care from the Animal Welfare Act and the NIH Guide for the Care and Use of Laboratory Animals. Transgenic VGAT-ChR2–EYFP mice were used for CBV-weighted fMRI with cortical inactivation (*n* = 22) and conventional CBV-weighted rs-fMRI (*n* = 4) and electrophysiological recordings (*n* = 6). One VGAT-ChR2 mouse and one VGAT–ChR2 negative littermate were used for histology. Naïve C57BL/6 mice were used to optimize imaging protocols for CBV-weighted fMRI (*n* = 3) and to study light-induced heating effects (*n* = 3) and conventional CBV-weighted rs-fMRI (*n* = 5; for comparison with cortical silencing fMRI). All fMRI experiments were carried out at an ultra-high field of 15.2 T for enhancing fMRI sensitivity (30, 76). The details of animal preparation, experiments, and data analyses are provided in *SI Appendix, Supplementary Methods*.

## Supplementary Material

Supplementary File

## Data Availability

All study data are included in the article and/or supporting information. The imaging data that support the findings are available at XNAT Central (https://central.xnat.org/data/projects/OS-fMRI).
