# Supplementary material for: Dissection of brain-wide resting-state and functional somatosensory circuits by fMRI with optogenetic silencing
Source: Proc Natl Acad Sci U S A. 2022 Jan 18;119(4):e2113313119. doi: 10.1073/pnas.2113313119 (PMC8795561; doi:10.1073/pnas.2113313119)
Supplement: Supplementary File [file pnas.2113313119.sapp.pdf]

Supporting Information (Methods and Figures)

Dissection of brain-wide resting-state and functional somatosensory circuits by fMRI with optogenetic silencing

Won Beom Jung[a], Haiyan Jiang[a,b], Soohyun Lee[c], and Seong-Gi Kim[a,b,d *]

[a] Center for Neuroscience Imaging Research (CNIR), Institute for Basic Science (IBS), Suwon 16419, Republic of Korea

[b] Department of Biomedical Engineering, Sungkyunkwan University, Suwon, 16419, Republic of Korea

[c] Unit on Functional Neural Circuits, National Institutes of Health, Bethesda, MD 20892, USA

[d] Department of Intelligent Precision Healthcare Convergence, Sungkyunkwan University, Suwon, 16419, Republic of Korea

***Corresponding author:**

Seong-Gi Kim, IBS Center for Neuroscience Imaging Research, N Center

Sungkyunkwan University, Suwon 16419, Republic of Korea

Email: seonggikim@skku.edu

Phone number: +82-31-299-4350

This file includes:

Supplementary text: Supplementary Methods

Figures S1 to S9

Tables S1 to S3

Legends for dataset S1

SI References

Other supplementary materials for this manuscript include the following:

Dataset S1

Supplementary methods

Animal preparation

A total of 45 mice (21.4–31.5 g, 6–13 weeks old, male/female = 36/9) were used in six different studies: 1) 22 transgenic mice for fMRI with cortical inactivation (VGAT-ChR2-EYFP, n=8 for S1FL, n=7 for M1, and n=7 for S2), 2) 6 VGAT-ChR2 mice for electrophysiology recording, 3) 3 naïve mice for contrast agent dose-dependent MRI to optimize the CBV-weighted fMRI protocol (C57BL/6; Orient Bio, Seongnam, Korea), 4) 3 naïve mice for the fMRI study on light-induced heating effects, 5) 5 naïve and 4 VGAT-ChR2 mice for conventional resting-state CBV-weighted fMRI, and 6) 1 VGAT-ChR2 positive mouse and 1 VGAT-ChR2 negative littermate for histological confirmation of GABAergic interneuron-specific expressions of ChR2 in transgenic VGAT-ChR2-EYFP mice. The VGAT-ChR2-EYFP mice (B6.Cg-Tg (Slc32a1-COP4*H134R/EYFP) 8Gfng/J) were bred in-house from breeding pairs obtained from Jackson Laboratory (Bar Harbor, ME, USA). Animals were housed in independently ventilated cages under controlled temperature and humidity conditions and a 12-hour dark-light cycle.

A stereotactic surgical procedure (1) was performed to implant fiber-optic cannulas (Thorlabs, Newton, New Jersey, USA) in mice (VGAT-ChR2-EYFP, n=22; C57BL/6, n=3) at 6-7 weeks of age for optogenetic fMRI. In short, the mice were anesthetized by an intraperitoneal (IP) bolus injection of a ketamine and xylazine mixture (100 mg/kg and 10 mg/kg, respectively) and fixed in a stereotaxic frame (SR-AM, Narishige, Tokyo, Japan) for surgery. Meloxicam (1 mg/kg) was administered subcutaneously to provide pain relief and reduce inflammation. After an incision was made on the animal's scalp, the skull was completely washed and dried. The skull was then thinned over the target area to make a burr hole (~ 100 µm diameter). The optical fiber cannula (105 µm inner core diameter, NA = 0.22) was then inserted slowly into the right S1FL (AP: - 0.2 mm relative to bregma, ML: + 2.2 mm and DV: + 0.5 mm relative to the surface), M1 (AP: + 0.05 mm, ML: + 1.1 mm and DV: + 0.25 mm) and S2 (AP: - 1.1 mm, ML: + 4.2 mm and DV: + 0.9 mm) areas (see Fig. S2A for visualization of tip position) through the burr hole at a rate of 1 µm/sec via a

micromanipulator (SMX-model, Sensapex, Oulu, Finland). With the fiber-optic implant in place, a biocompatible silicone elastomer (Kwik-Sil, World Precision Instruments, Sarasota, FL, USA) was applied to enclose the fiber implantation site, and dental cement (SB, Sun-Medical Co., Shiga, Japan) was then applied to thinly cover the area around the fiber cannula to fix it onto the skull. After a recovery period of at least 2 weeks, mice underwent optogenetic fMRI experiments.

The functional experimental procedure was previously described in detail (2, 3). In short, mice were anesthetized using a mixture of ketamine (Yuhan, Korea) and xylazine (Rompun®, Bayer, Korea) (100/10 mg/kg for initial IP, and 25/1.25 mg/kg intermittent IP injections every 45-50 min) under self-breathing through a nose cone that provided a continuous supply of oxygen and air (1:4 ratio) at a rate of 1 liter/min (SAR-1000, CWE, Ardmore, USA or TOPO, Kent Scientific Corporation, Torrington, CT, USA) to maintain an oxygen saturation level > 90% (4). During the experiments, electrocardiograms and motion-sensitive respiratory signals were continuously monitored (Model 1030, Small Animal Instruments Inc., Stony Brook, USA for fMRI experiments and PhysioSuite, Kent Scientific Corp, USA for electrophysiology studies). Also, the body temperature of the animals was maintained at 37 ± 0.5 °C with a warm-water heating system and a rectal thermometer.

Stimulation

The stimulation parameters were controlled by a pulse generator (Master 9; World Precision Instruments, Sarasota, FL, USA). For light stimulation, blue light was delivered to the target cortex via a fiber-optic cable coupled to a 473-nm diode-pumped solid-state laser (MBL-III-473, Changchun New Industries Optoelectronics Tech. Co., Ltd, Changchun, China). The constant output power was calibrated to be 3 mW at the tip of the optic fiber, measured by a power meter (PM100D, Thorlabs, USA). Light stimulation was then applied at 20 Hz with a pulse width of 10 ms via a 105 μm diameter fiber. This experimental setting leads to a time-averaged light power of

69.3 mW/mm^2, which is below the range at which light-induced artifactual responses are generated (5, 6). To block undesired activation of the visual pathway by light leakage inside the magnet bore, the connection between the fiber-optic cable and the implanted cannula was covered with heat-shrinkable sleeves, and the eyes of the animals were covered with a biocompatible silicone elastomer. For somatosensory stimulation, the left forepaws of the mice were electrically stimulated. A pair of needle electrodes (30 G) were inserted under the plantar skin of the forepaws, and electrical pulse stimuli with a current intensity of 0.5 mA (ISO-Flex, AMPI, Jerusalem, Israel), pulse width of 0.5 ms, and frequency of 4 Hz were triggered by a pulse generator (2). Each functional trial consisted of a 60-s (30 s for electrophysiology) prestimulus, 20-s stimulus, 60-s interstimulus interval, 20-s stimulus, and 60-s (30 s for electrophysiology) poststimulus period.

Electrophysiology

Multiunit activity (MUA) and local field potential (LFP) were measured in the S1FL to confirm the suppression of neuronal activity during optogenetic cortical inactivation and to investigate the neural source of fMRI findings concerning long-range TC input and local recurrent activity (VGAT-ChR2-EYFP, n=6). The head was fixed in a stereotaxic frame (SR-10R-HT, Narishige, Tokyo, Japan), and the scalp was removed to expose the skull. After cleaning the skull, two holes (> 0.3 mm in diameter) were made with a dental drill to implant the stainless-steel screws for the ground and reference wires. Craniotomy was performed over the right somatosensory area, with a diameter of ~2 mm. CBV-weighted optical imaging (Imager 3001, Optical Imaging Ltd., Rehovot, Israel) of the responses to left forepaw stimulation (FP) was performed to precisely determine the S1FL area. To simultaneously record neural activity during optogenetic stimulation, a 16-channel optoelectrode with 50 μm spacing between channels (A1x16-5 mm-50-177-OA16LP, NeuroNexus, Ann Arbor, MI, USA) was placed at the center of the predefined S1FL and slowly inserted at a speed of 1 μm/sec. The fiber tip in this optoelectrode was positioned ~ 200 μm above the proximal recording site nearest to the cortical surface. In one mouse, a separate optic fiber was inserted into

the middle of the cortex obliquely near the optoelectrode to determine optogenetic cortical inactivation across all cortical depths (see Fig. S2B-i for visualization of the tip position). During insertion, the electrode was held for 5 min every 100 μm and for 10 min when the tip of the electrode reached a cortical depth of ~1 mm to stabilize the position. Electrophysiological signals were measured at a sampling rate of 30 kHz using a Blackrock Cerebus recording system (Blackrock Microsystems, Salt Lake City, Utah). Functional studies were interleaved across 3 different stimulus paradigms, and 5-8 trials were performed for each experimental condition for each animal.

MRI experiments

All MRI experiments were performed on a 15.2 T MRI scanner with an actively shielded 6-cm diameter gradient operating with a maximum strength of 100 G/cm and a rise time of 110 μs (Bruker BioSpec, Billerica, MA, USA). A 15 mm ID customized surface coil for radiofrequency (RF) transmission/reception was centered on the imaging slices covering the somatosensory cortex, and the brain was positioned close to the isocenter of the magnet. Magnetic field homogeneity was optimized by global shimming, followed by the FASTMAP shimming protocol on the ellipsoidal volume covering the cerebrum (ParaVision 6, Bruker BioSpin).

Calibration of the amount of contrast agent used for CBV-weighted fMRI studies. Gradient-echo (GE) echo planar imaging (EPI), commonly used in functional studies, is sensitive to susceptibility artifacts arising from implanted optic fibers, particularly at long TEs and high field strengths (Fig. S3A). Importantly, the BOLD contrast measured by GE-EPI is highly sensitive to draining veins, resulting in poor specificity for cortical layers. CBV-weighted fMRI with a superparamagnetic contrast agent is an alternative method to GE-BOLD to improve the functional sensitivity and spatial specificity at neuronal active sites (7). CBV-weighted fMRI with a short TE has the advantage of reducing nonspecific BOLD contributions and minimizing susceptibility artifacts.

Initially, we measured the relationship between contrast doses and T_2^* values by repeated injections of monocrystalline iron oxide nanoparticles (MIONs; Feraheme, AMAG Pharmaceuticals, Waltham, USA) into the tail vein until the cumulative dose reached 45 mg/kg (from 2 to 45 mg/kg) in 3 naïve mice (Fig. S3B). T_2^*-weighted images were obtained using the multi-gradient-recalled echo (Multi-GRE) sequence with sixteen echo times = 1.5 to 18 ms and an interval of 1.1 ms, repetition time (TR) = 0.8 s, field of view (FOV) = 15 (read-out, x-axis) × 7.5 (phase-encoding, y-axis) mm^2, matrix = 96 × 48, spatial resolution = 156 × 156 × 500 μm^3, flip angle = 50°, 6 contiguous coronal slices and number of excitations (NEX) = 4.

fMRI combined with optogenetics. Our imaging strategy was to use a very short TE for fMRI data without susceptibility artifacts while maximizing the functional sensitivity. Therefore, TE was set to 3 ms for matching to tissue T_2^* (3.44 ± 0.08 ms in Fig. S3B) values, with MION at a 45 mg/kg dose. CBV-weighted fMRI data were acquired using the fast low-angle shot magnetic resonance imaging (FLASH) sequence with the following parameters: TR/TE = 50/3 ms, flip angle = 15°, FOV = 15 × 7.5 mm^2, matrix size = 96 × 48, partial Fourier = 1.2 along the phase-encoding direction, spatial resolution = 156 × 156 × 500 μm^3, 6 contiguous coronal slices and temporal resolution = 2 s. From individual transgenic mice (n=22 mice), fMRI data were acquired under three stimulus conditions: optogenetic cortical silencing (n=8 for S1FL targeting, n=7 for M1 targeting, and n=7 for S2 targeting), FP, and simultaneous silencing and FP. Data for the three stimulus conditions were acquired in an interleaved manner, with an interscan interval longer than 3 min. To address the potential heating-related artifacts induced by light, functional studies in naïve mice (n=3) were interleaved between optogenetic and FPs. For each stimulus condition, 15 fMRI trials were performed for each animal.

rs-fMRI. Conventional rs-fMRI, which is presumably related to the intrinsic network of spontaneous activity, was obtained to examine the resting-state somatosensory network. Ten 10-min rs-fMRI scans (i.e., 300 volumes) were obtained from two separate animal groups, naïve

animals (n=5, twice each) and transgenic VGAT-ChR2 animals (n=4, twice each) with the same imaging parameters used for fMRI, after a few somatosensory fMRI scans to ensure the physiological condition of the mice responding to external stimuli under anesthesia.

Histology

To confirm GABAergic interneuron-specific expression of ChR2 in home-bred transgenic mice, we performed histology of one VGAT-ChR2-EYFP positive mouse and one VGAT-ChR2-EYFP negative littermate mouse. Animals were anesthetized with ketamine/xylazine and intracardially perfused with saline, followed by 4% paraformaldehyde (PFA) for fixation. The brains were postfixed in 4% PFA overnight at 4 °C and then stabilized with 30% sucrose with 0.1% sodium azide solution at 4 °C for 3 days. The brains were coronally sectioned at a 40-μm thickness with a cryostat (CM 1950, Leica Biosystems). Two sections were selected to check ChR2 expression: one included the S1FL to validate the expression of ChR2 in cortical GABAergic neurons, and the other included the GABAergic neuron-rich thalamic reticular nucleus (TRN). The sections were incubated with mouse anti-GAD67 primary antibodies (1:300; Millipore) and decorated with secondary antibodies conjugated with Alexa 568 (1:350; Invitrogen) to label interneurons. Colocalization of GAD67-positive neurons with EYFP expression in transgenic mouse indicated that ChR2 was specific to GABAergic neurons. Nuclear counterstaining was also performed with DAPI solution (1:10,000; Sigma) in PBS. The stained brain slices were imaged with confocal laser microscopy (TCS SP8, Leica Microsystems). The green-, red-, and blue-colored representations depict the relative spectral weights for ChR2-EYFP-, GAD67- and DAPI-positive expression, respectively.

Data analysis

Electrophysiology data analysis. To detect spiking activity, the extracellular recording traces were preprocessed as follows: first, raw data were high-pass filtered (> 300 Hz; 4th-order Butterworth

filter); second, the common noise level was temporally removed by subtracting the trimmed mean (50%) filtered signals over all channels at each time point from each channel; third, MUA was quantified over 1 ms temporal bins by counting the number of spikes exceeding a threshold of 5 × the median absolute deviation in 30 s of prestimulus baseline data (8). The LFP data were extracted from the extracellular recording traces by bandpass filtering at 1 to 280 Hz and downsampled to 1 kHz. For the comparison between trials with FP at 4 Hz and optogenetic silencing at 20 Hz, spike trains and LFP waveforms during stimulation were aligned with a 250-ms moving window after the first stimulus onset of each block, and repeated runs (20 s × 2 blocks × 4 windows/s × # of trials) were then averaged (Fig. S8B).

To confirm the suppression of spontaneous neural activity during optogenetic cortical stimulation, each light pulse-induced putative inhibitory neural activity was excluded for the 10 ms light pulse period and followed up for 10 ms, resulting in spontaneous spike activity during the 30 ms period following each 20 Hz stimulation pulse (9). The firing rates were also calculated across the upper 8 and lower 8 channels to measure depth dependency.

To investigate the cortical depth-dependent MUA and LFP responses, we first performed inversion current source density (iCSD) analysis with the LFP waveform using the CSDplotter toolbox (10) to identify the location of L4. The synaptic inputs induced by FP were observed as a strong sink at depths of 0.4-0.56 mm, whereas the current sink generated by each optogenetic stimulation was shown at depths of 0.2-0.4 mm (Fig. S8C). Based on these laminar CSD profiles, the cortical depth of the S1FL was assigned as L2/3, L4 and L5/6 (Fig. S8D and S8E).

We compared the spiking responses and field potential observed during optogenetic silencing without and with FP. Since somatosensory-evoked recurrent excitatory circuits were suppressed by optogenetically activating inhibitory neurons in the S1FL, and the remaining activity indicated direct feedforward inputs from the thalamus ($Diff_{S1FL}$). The contribution of silenced local recurrent circuits was calculated by the difference between TC input-driven activity and the total somatosensory-evoked response. To alleviate minor response timing errors from the calculation,

three sensory-evoked MUA traces and LFP waveforms (TC input-driven activity vs. local recurrent activity vs. total activity) were fitted using a single Gaussian function and double gamma variate function, respectively, averaged across all channels and for each animal.

In the fitted MUA curve, activity parameters, including peak amplitude and the area under curve (AUC), and dynamic parameters, including onset time, peak time, and full-width at half-maximum (FWHM), were quantified. Response onset was defined as the first bin of five continuous bins in which the spike rate differed significantly from that observed at baseline activity ($p < 0.05$, one-sample t-test). In LFPs, the same parameters described for MUA were calculated except for the onset time.

Calibration of the amount of contrast agent. To determine MION dose-dependent tissue T_2^*, multi-GRE data before and after every injection were fitted by a monoexponential function as $S_0 e^{-TE/T_2^*}$, where S_0 is the signal at TE = 0 ms. For quantification of tissue T_2^* changes, the ROI was defined as a two-dimensional (2D) sphere with a radius of 500 μm centered on the anatomical location of the S1FL with reference to the Allen mouse brain atlas.

Overall fMRI data analysis. All MRI data were analyzed with the Analysis of Functional Neuroimages package (AFNI) (11), FMRIB Software Library (FSL) (12), Advanced Normalization Tools (ANTs) (13), and MATLAB codes (MathWorks, Natick, USA).

To calculate group-averaged fMRI maps, a study-specific mouse brain template was constructed from CBV-weighted fMRI images based on a previously used pipeline (14, 15) as follows. First, the brain area was semiautomatically extracted from the temporal average of all fMRI images for each animal, and intensity nonuniformity was corrected with bias field estimation. Second, the individual fMRI images were aligned with each other so that the most representative image requiring the least transformation within the population could be selected (12). Third, all the images were spatially normalized into that representative image using both linear and nonlinear

transformation and averaged to generate the mouse brain template. To identify the anatomical location, the study-specific template was coregistered with the Allen mouse brain atlas (16).

Generation of evoked-fMRI maps. The fMRI response maps for individual animals were generated using preprocessing and general linear model (GLM) analysis. The preprocessing steps were previously described in detail (3, 14). In short, preprocessing included slice timing correction, image realignment for minor head motions, linear signal detrending for signal drift removal, and time course normalization by the average of the baseline period. All repeated fMRI trials per stimulus condition for each animal were averaged and spatially coregistered to study-specific brain templates. Then, spatial smoothing was applied with a Gaussian kernel of one-pixel FWHM to minimize potential misalignment and to enhance functional detectability. Individual fMRI maps were calculated by a GLM analysis with design matrices where two 20-s stimulus blocks were convolved by the data-driven hemodynamic response function (HRF). The HRF was determined from the animal-wise averaged time course in the S1FL during FP by fitting with a two-gamma variate function. After GLM analysis, group-averaged fMRI maps of FP, optogenetic stimulation only (Opto), and combined forepaw and optogenetic stimulation (Comb) were generated for the S1FL, M1 and S2 silencing conditions with a one-sample t-test using nonparametric inference (12), with significance at threshold-free cluster enhancement (TFCE)-corrected $p < 0.05$. Standardized regression coefficients (β value) were mapped.

To determine somatosensory input-driven responses without the feedforward or local contribution from the inactivation site, fMRI time courses during optogenetic cortical inactivation with and without FP were subtracted (Comb – Opto), and difference maps (Diff) were obtained with a significance level of uncorrected $p < 0.01$ (paired t-test). In addition, all animal data (n=22) were used to generate group-averaged FP activation maps. The group-averaged fMRI maps were overlaid on the study-specific brain template, and the fMRI active sites were determined according to the Allen mouse brain atlas.

Quantitative ROI analyses for circuit analysis. For quantitative analysis, five different somatosensory ROIs corresponding to active sites during FP were defined based on the Allen mouse brain atlas: S1FL, M1, S2, VPL, and POm. Time courses from each ROI were obtained for FP, Opto, Comb, and Diff conditions in each animal, and fMRI signal changes were calculated by averaging responses over the stimulus condition, excluding the initial 6 s after stimulus onset to obtain steady-state positive or negative responses without the initial transition period. An increase in CBV increased the amount of iron oxides within the voxel, consequently decreasing fMRI signals. Thus, the original CBV-weighted fMRI signal changes were inverted for better visualization in all analyses.

Seed-based rs-fMRI mapping. For rs-fMRI data without any stimulations, preprocessing steps similar to those used for evoked fMRI were included: slice timing correction, image realignment, linear detrending, and voxel-wise time course normalization. To stabilize functional connectivity, additional preprocessing steps were applied: spiking signal removal, data cleaning by regressing out nuisance variables including 12 motion confounds (6 parameters + temporal derivatives) and brain global signals, bandpass filtering ($0.01 < f < 0.2$ Hz) (2) and spatial smoothing with a Gaussian kernel of a 0.5 mm FWHM. Three seed ROIs of S1FL, M1 and S2 in the right hemisphere were anatomically defined based on the Allen mouse brain atlas. To identify the resting-state connectivity dependent on mouse strain, rs-fMRI data from 5 naïve mice (Fig. 2B) and from 4 transgenic mice (Fig. S4A) were separately analyzed. In common space, functional connectivity maps for individual animals were calculated by correlation analysis of the mean time courses of each seed ROI and voxel-wise time series in the whole brain. To generate group-averaged rs-fMRI maps, voxel-wise correlation coefficient (r) was converted to a normally distributed z-score using Fisher's r-to-z transformation, and a one-sample t-test was performed (TFCE-corrected $p < 0.05$). After statistical testing, the results were inversely transformed back to correlation coefficients to represent group-averaged rs-fMRI maps.

Resting-state connectivity analysis. To investigate spatial patterns of spontaneous somatosensory network, we generated the two different connectivity maps with 1) cross-correlation coefficients (r) of rs-fMRI using temporal correlation in S1FL, M1, and S2 seed-regions (see subsection of *seed-based rs-fMRI mapping*) and 2) regression coefficients (β value) of fMRI with cortical silencing in S1FL, M1, and S2 areas using GLM analysis (Opto) (see subsection of *generation of evoked-fMRI maps*). The degree of rs-fMRI correlation between two different regions often considers as a proxy to spontaneous neural network strength (17), while fMRI signal changes in the networked areas causally induced by cortical silencing is related to the degree of interregional communication with source area under basal conditions (18). To compare the relative strength of spontaneous functional connectivities measured by rs-fMRI and cortical silencing fMRI (Opto), 1) the magnitude of functional connectivity (z-scores by Fisher's r-to-z transform) within predefined somatosensory ROIs was normalized to the strongest connectivity with a seed area (given a maximum z-value equal to 1) in rs-fMRI data of naïve mice, 2) whereas the fMRI change in spontaneous connectivity during cortical silencing fMRI (Opto) was normalized to the response at the optogenetic stimulation site in VGAT-ChR2 mice. To examine which functional connectivity patterns closely reflect the intrinsic neural networks, tracer-based connectivity maps for S1FL (experiment #112229814), M1 (experiment #100141563) and S2 injection (experiment #112514915) were obtained from the Allen Institute (16). The connectivity strengths in networked areas were normalized by the projection density in the injection site (Fig. S4B).

Layer analysis of fMRI responses. To measure synaptic input layer-specific fMRI responses, all aforementioned preprocessing steps except spatial smoothing were applied. Cortical areas, including the S1FL, M1, and S2, were flattened by radially projecting 22 lines perpendicular to the cortical edges (19). The cortical depth profiles were resampled to double using bicubic interpolation, leading to a nominal resolution of 78 μm. Then, in each animal, an averaged profile was obtained in the S1FL and M1 by averaging 5-pixel lines (0.78 mm width) and in S2 by averaging 10-pixel

lines (1.56 mm width). Group-averaged percent change maps were calculated from the percent change maps of individual animals. The fMRI signal changes within the same cortical depth were calculated in the same manner described above. Laminar boundaries for each cortex were defined as a cortical thickness distribution based on the Allen mouse brain atlas.

Statistics. All quantitative values are presented as the mean ± standard error of the mean (SEM). For a comparison of the suppression of spontaneous MUA firing rates with and without optogenetic inactivation (Fig. 1B-ii), an evaluation of fMRI signal changes for measuring heating-related fMRI artifacts (Fig. 1D), a comparison of fMRI signal changes between long-range sensory inputs (i.e., difference between cortical inactivation with and without FP) and total sensory-evoked activity (FP only) (Fig. 3C, 3D, 4C, 4D and 6B), and the analysis of the relative fraction of long-range inputs and local recurrent activities in the MUA and LFP (Fig. 4G), a paired t-test was conducted to determine the statistical significance of differences. For a comparison of fMRI signal changes across somatosensory fMRI experiments in different animal groups, one-way analysis of variance (ANOVA) was conducted (Fig. 3C, 3D, 4C, 4D and 6B). To compare the cortical depth-dependent MUA and LFP data (Fig. S8F-H), one-way ANOVA with repeated measures was performed. All ANOVA tests were followed by the Bonferroni *post hoc* test for multiple comparisons. Statistical significance was considered at $p < 0.05$.

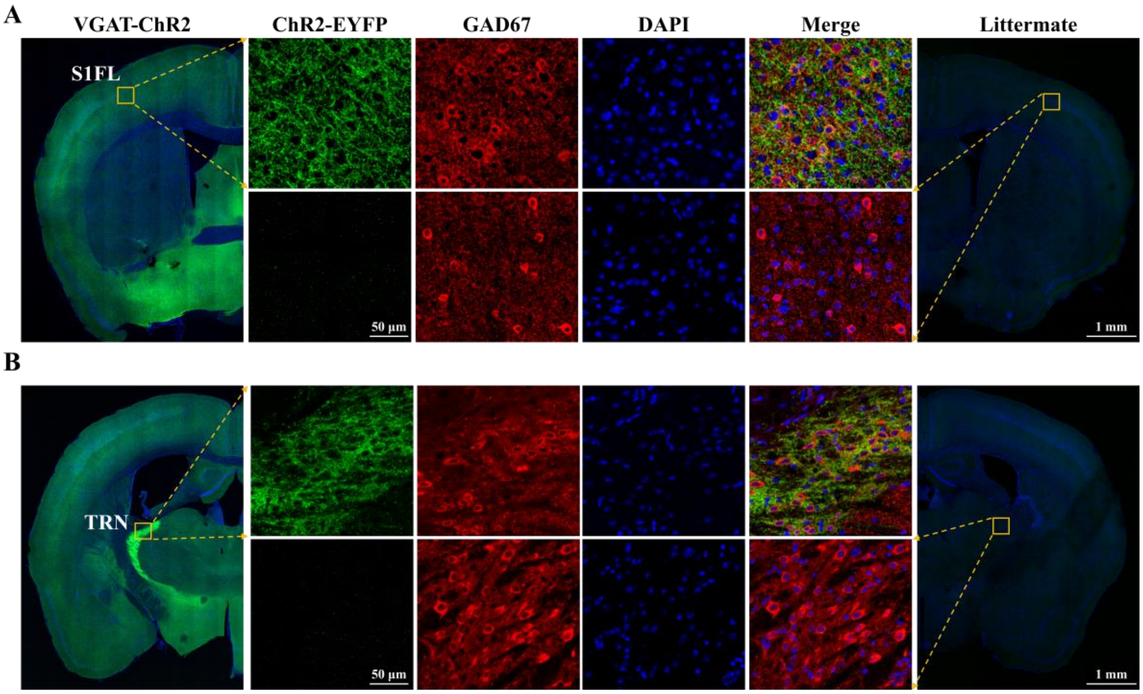

Figure S1. GABAergic interneuron-specific expression of ChR2-EYFP in VGAT-ChR2 transgenic mouse.

(A-B) Transgenic-specific expression of ChR2-EYFP in **(A)** the primary somatosensory cortex and **(B)** the GABAergic neuron-rich thalamic reticular nucleus of a VGAT-ChR2 positive mouse (the leftmost column and top panel) vs. that of a VGAT-ChR2 negative littermate mouse (the rightmost column and bottom panel). One S1FL-containing slice was chosen to validate the expression of ChR2 in cortical GABAergic neurons. Colocalization of ChR2-EYFP (green) and interneuron markers (GAD67, red) indicates that ChR2 is specific to GABAergic neurons. DAPI was used to visualize cell nuclei (blue). Our histology data are consistent with the reported expression of this transgenic mouse line (20).

Green-, blue-, and red-colored representation of i, ChR2-EYFP, DAPI and DiI fluorescence, respectively.

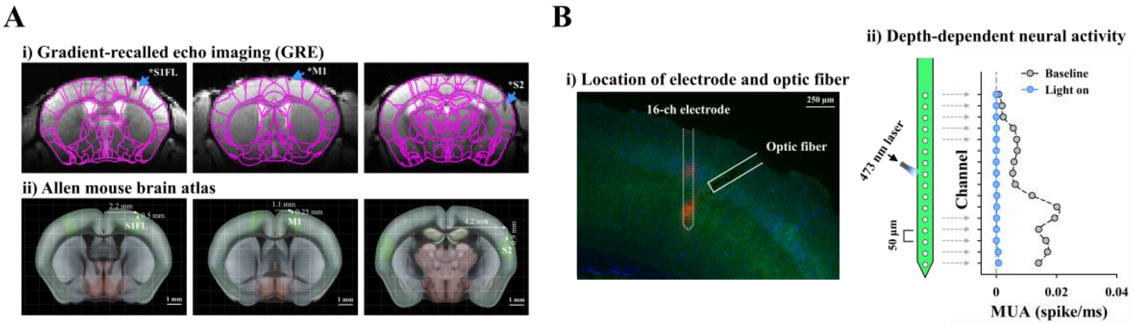

Figure S2. Fiber position and depth-dependent neural activity in the S1FL

(A) High-resolution mouse brain MR images with fiber-optic implants in the S1FL, M1, and S2 and the corresponding Allen brain atlas. The fiber tip (dark line indicated by a blue arrow) was inserted into the middle of the cortex in the S1FL (0.5 mm from the cortical surface) and S2 and the upper cortical area in M1 (0.25 mm from the cortical surface).

(B) Depth-dependent electrophysiological data in the S1FL without (baseline, gray profile) and with optogenetic stimulation (light on, blue profile) of VGAT-ChR2 transgenic mouse. The 16-channel recording electrode with 50 µm spacing was inserted 1 mm deep, covering a depth between 200 and 1000 µm (dashed line in i with two red spots for electrode position marking), while a 105 µm-diameter optic fiber was obliquely targeted an area 500 µm below the cortical surface (white lined bar in i) to mimic optogenetic fMRI studies in the S1FL. Blue light directly stimulated ChR2-expressing interneurons in the middle and lower cortical layers. However, the MUA was suppressed completely across entire cortical depths, including the upper cortical area above the fiber tip (ii; see also Fig. 1B with cortical surface illumination). This suggests that focal cortical inhibition is effective across all cortical depths, possibly due to recurrent excitatory circuits within a column.

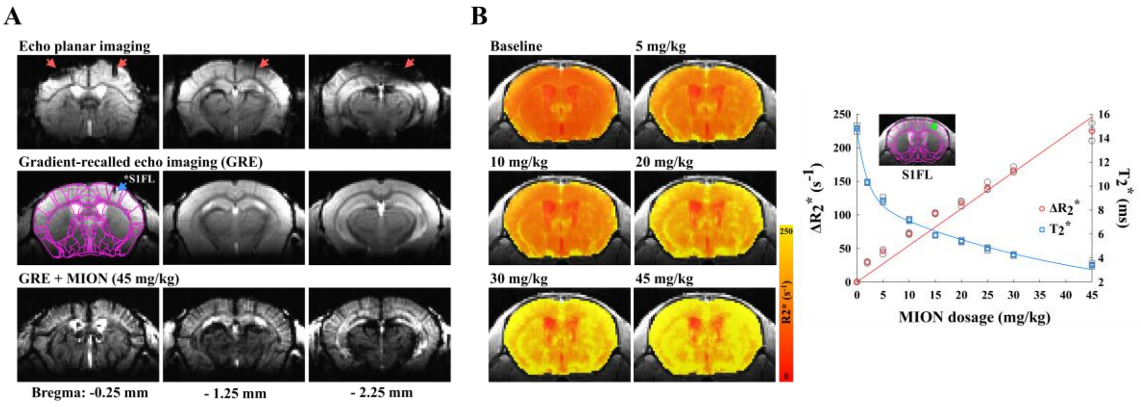

Figure S3. Imaging quality control for optogenetic CBV-weighted fMRI.

(A) High-resolution mouse brain MR images from one mouse with a fiber-optic implant. Magnetic susceptibility distortion and signal dropout (red arrows) in single-shot gradient-echo EPI images were minimized by adoption of gradient-recalled echo imaging with a short echo time of 3 ms (blue arrow, fiber position). To enhance functional sensitivity and specificity, a 45 mg/kg dose of the superparamagnetic monocrystalline iron oxide nanoparticle (MION) agent was injected into the animals' blood.

(B) Contrast agent dose-dependent transverse relaxation rate R_2^* $(= 1/T_2^*)$ changes. The R_2^* maps using multiple gradient-echo imaging (left) were acquired before and after cumulative doses ranging from 2 mg/kg to 45 mg/kg MION in the same animals (wild-type, n=3) to determine the optimal echo time matching to tissue T_2^* for CBV-weighted fMRI. Tissue T_2* (blue symbols) and ΔR_2^* (red symbols) were calculated from S1FL ROI at each MION dose.

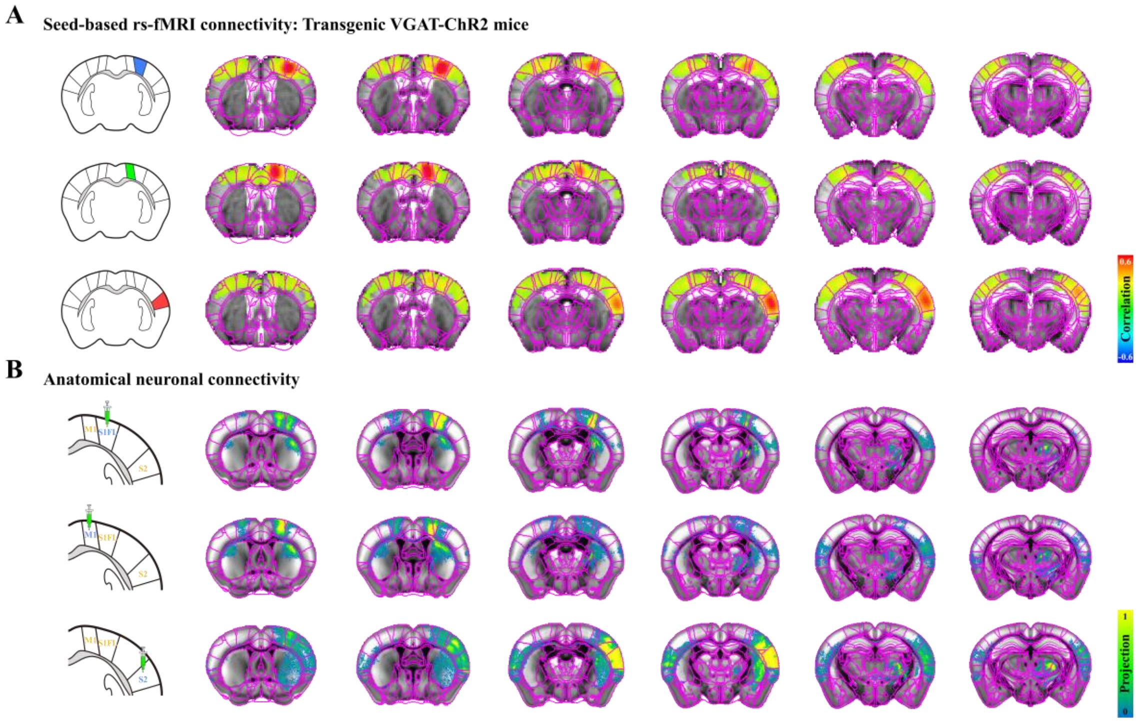

Figure S4. Bilateral homotopic rs-fMRI connectivity is not primarily mediated by direct neural corticocortical connections.

(A) Functional connectivity maps measured by seed-based rs-fMRI in VGAT-ChR2 mice. To determine whether bilateral homotopic correlation is a general feature of rs-fMRI independent of strain, functional connectivity was measured with the seed ROIs of S1FL, M1 and S2 in VGAT-ChR2 mice. Similar to the findings of naïve mice (Fig. 2B), resting-state fMRI in transgenic mice showed a strong correlation between bilateral somatosensory cortices but not between the cortex and thalamus in the ipsilateral hemisphere.

(B) Neural connectivity maps projected from somatosensory cortices, obtained from the Allen Institute (16). Ipsilateral projections in the somatosensory network are generally stronger than contralateral ones.

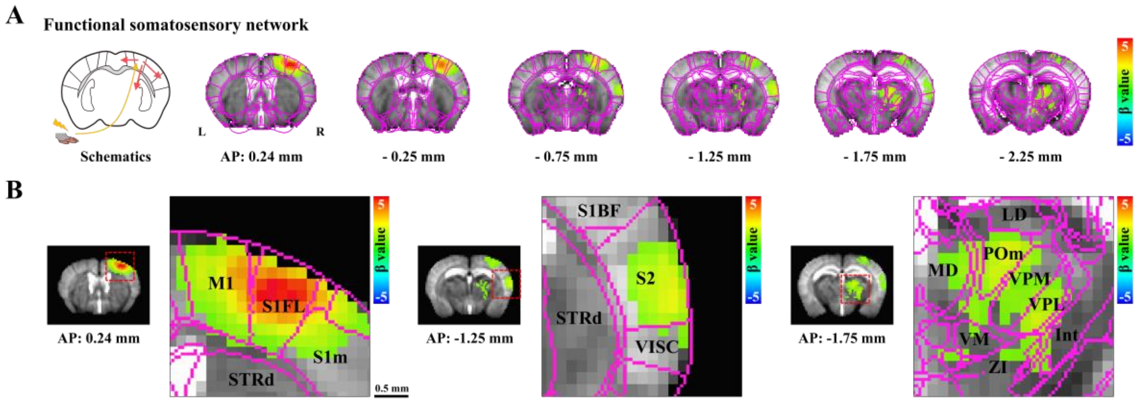

Figure S5. Brain-wide fMRI maps of somatosensory-evoked activities.

(A) Contralateral somatosensory regions, including the cortices (S1FL, M1 and S2) and thalamic nuclei (VPL and POm), responded to forepaw stimulation (VGAT-ChR2, n=22). Anterior-posterior slice coordinates relative to bregma are also marked. R, right; L, left hemisphere.

(B) Expanded fMRI maps in cortical and thalamic regions overlaid on the mouse brain atlas with labeling.

M1, primary motor area; S1FL, primary somatosensory area of forelimb; S1m, primary somatosensory area of mouth; STRd, striatum of dorsal region; S1BF, primary somatosensory area of barrel field; S2, secondary somatosensory area; VISC, visceral area; LD, lateral dorsal nucleus; MD, mediodorsal nucleus; POm, posterior medial nucleus; VPM, ventral posteromedial nucleus; VPL, ventral posterolateral nucleus; VM, ventral medial nucleus; Int, internal capsule; ZI, zona incerta; scale bar, 0.5 mm.

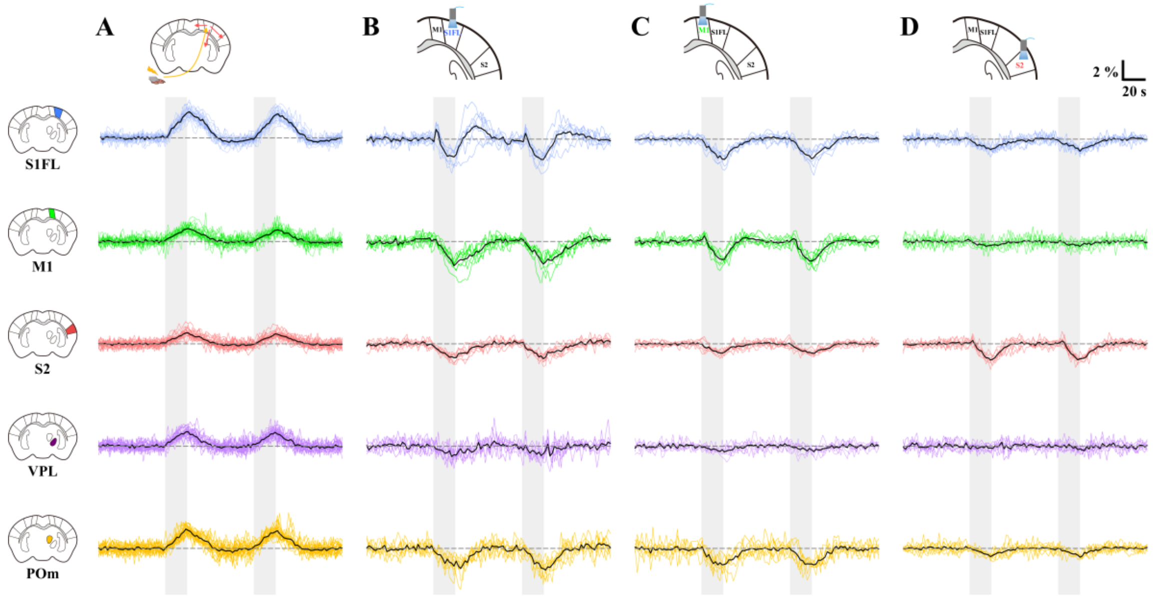

Figure S6. CBV-weighted fMRI responses of individual animals in the somatosensory network during forepaw stimulation and optogenetic cortical inactivation.

The fMRI time courses in the cortical (S1FL, blue lines; M1, green; S2, red) and thalamic (VPL, purple; POm, yellow) areas of individual animals during **(A)** forepaw stimulation, **(B)** S1FL inactivation, **(C)** M1 inactivation and **(D)** S2 inactivation were plotted. Black time course, animal-wise averaged response; gray vertical bar, 20-s stimulus.

One interesting observation is the CBV response at the optogenetically stimulating site: S1FL under S1FL Opto, M1 under M1 Opto, and S2 under S2 Opto. In the S1FL, an initial positive CBV response was followed by a negative CBV change and then a poststimulus positive CBV response. In S2, only negative CBV responses were observed. The initial positive CBV response was induced by activated inhibitory neurons, while the negative response was due to suppressed excitatory neurons. The underlying reason for different responses is unclear but could possibly be due to different combinations of active inhibitory vs. excitatory neurons in different regions. A large inter-animal variation was observed in the S1FL under S1FL Opto stimulation. This large variation was removed except for one animal when the difference between optogenetic stimulation with and without forepaw stimulation (Diff) was obtained. When we removed one outlier, the initial positive CBV response and the poststimulus overshoot disappeared in the averaged Diff time trace. However, these outlier data did not have much impact on an averaged trace (Fig. 4D $\text{Diff}_{\text{S1FL}}$).

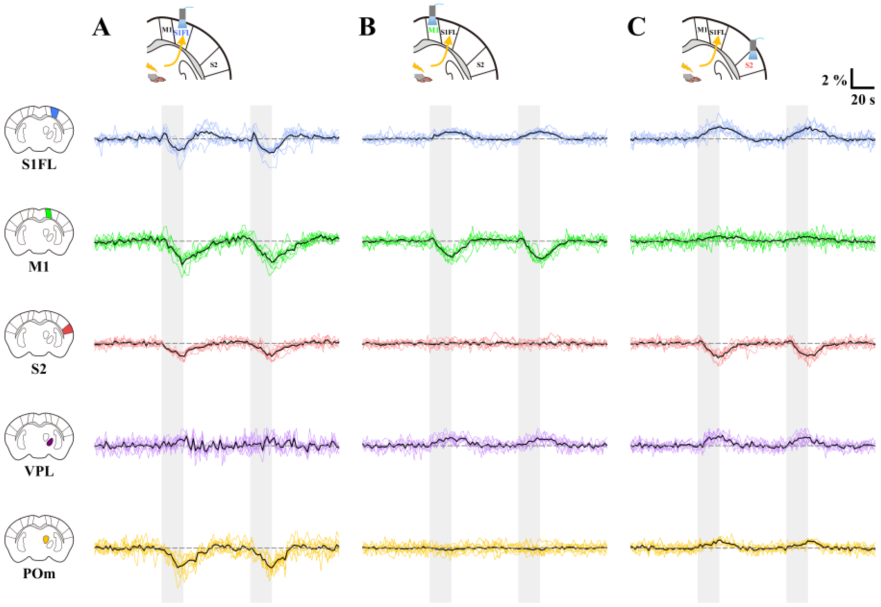

Figure S7. CBV-weighted fMRI responses of individual animals in the somatosensory network during simultaneous cortical inactivation and forepaw stimulation.

The fMRI time courses in the cortical (S1FL, blue lines; M1, green; S2, red) and thalamic (VPL, purple; POm, yellow) areas of individual animals during forepaw stimulation with **(A)** S1FL inactivation, **(B)** M1 inactivation and **(C)** S2 inactivation were plotted. Black time course, animal-wise averaged response; gray vertical bar, 20-s stimulus.

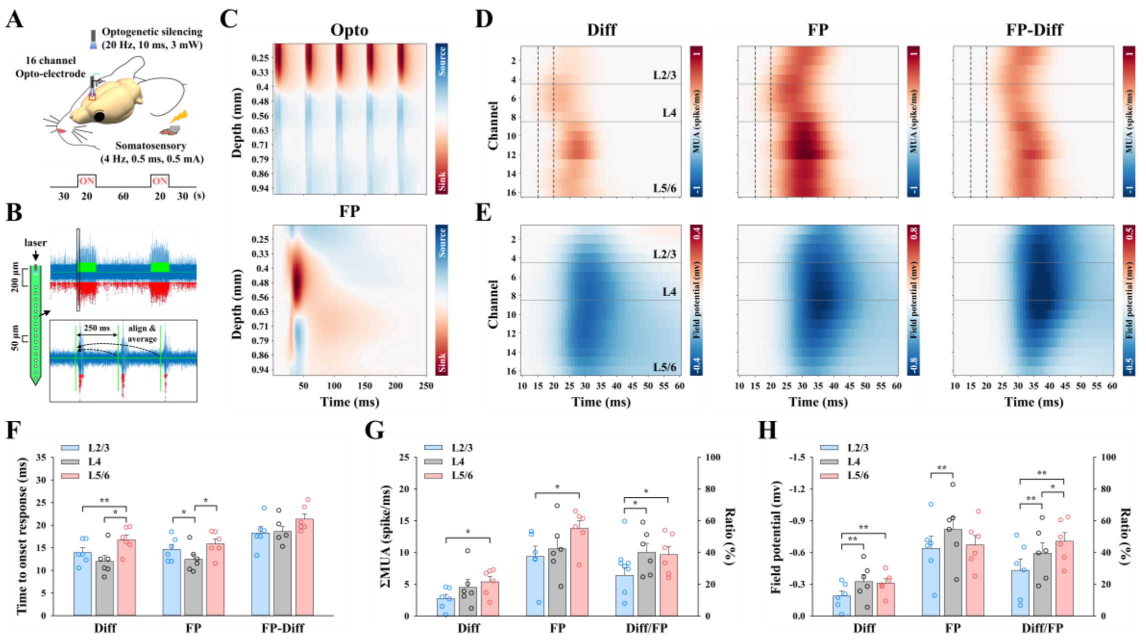

Figure S8. Cortical depth-dependent MUA and LFPs to distinguish sensory input-driven activity and local recurrent activity.

(A) Experimental schemes of electrophysiological recording with the 16-channel optoelectrode to measure somatosensory-evoked neural activity during S1FL inactivation (VGAT-ChR2, n=6). Optogenetic stimulation was performed on the surface of the S1FL cortex.

(B) Quantification of neuronal activity changes during stimulation. An example MUA trace with 4 Hz forepaw stimulation recorded from a single channel is shown (raw and expanded traces). The traces during two 20-s stimulus periods were aligned with a 250-ms window for comparison between neural activities acquired from 4 Hz somatosensory and 20 Hz optogenetic silencing. Spike activities from repeated MUA trains (or field potential from LFP waveforms) were then averaged across 20-s duration × 2 blocks × 4 windows/s × # of trials. Green line, forepaw stimulus duration; red dot, detected multiunit spike.

(C) Current source density (CSD) depth profile to identify the location of layer 4 (L4). Current sinks induced by optogenetic stimulation were found at depths of 0.2-0.4 mm below the cortical surface, whereas sink activity induced by forepaw stimulation was observed at depths of 0.4-0.56 mm (n=1), which is defined as L4. Red-to-blue, current sink-to-source.

(D-E) Time-dependent MUA and LFP depth profile in the S1FL (n=6) responding to somatosensory stimulation. **(D)** MUA trains and **(E)** LFP waveforms for the difference between

optogenetic silencing and silencing/forepaw stimulation (Diff; input-driven) and for forepaw stimulation (FP) were compared to distinguish the local recurrent activity (FP-Diff). The earliest MUA response to forepaw stimulation occurred at L4, which is expected from thalamocortical inputs, and recurrent activity observed ~6 ms later. Gray lines, borders of the S1FL layer; black dashed lines in **(D)**, time periods of 15 and 20 ms after stimulus onset.

(F) Cortical depth-dependent neural onset time. Somatosensory input-driven MUA (Diff) was first evoked at L4 of the S1FL approximately 12 ms after stimulus onset, but local recurrent activity (FP-Diff) occurred similarly across the layers.

(G-H) Cortical depth-dependent neural strength and ratio of input-driven activity to total somatosensory-induced activity. **(G)** The MUA response peaked at L5/6, but **(H)** the LFP amplitude was stronger at L4. Thalamic input-driven activity was relatively lower in the upper cortical layers (blue bars) than in the granular and infragranular layers (gray and red bars). $^*p < 0.05$ and $^{**}\ p < 0.01$ (one-way analysis of variance with repeated measures followed by the Bonferroni *post hoc* test).

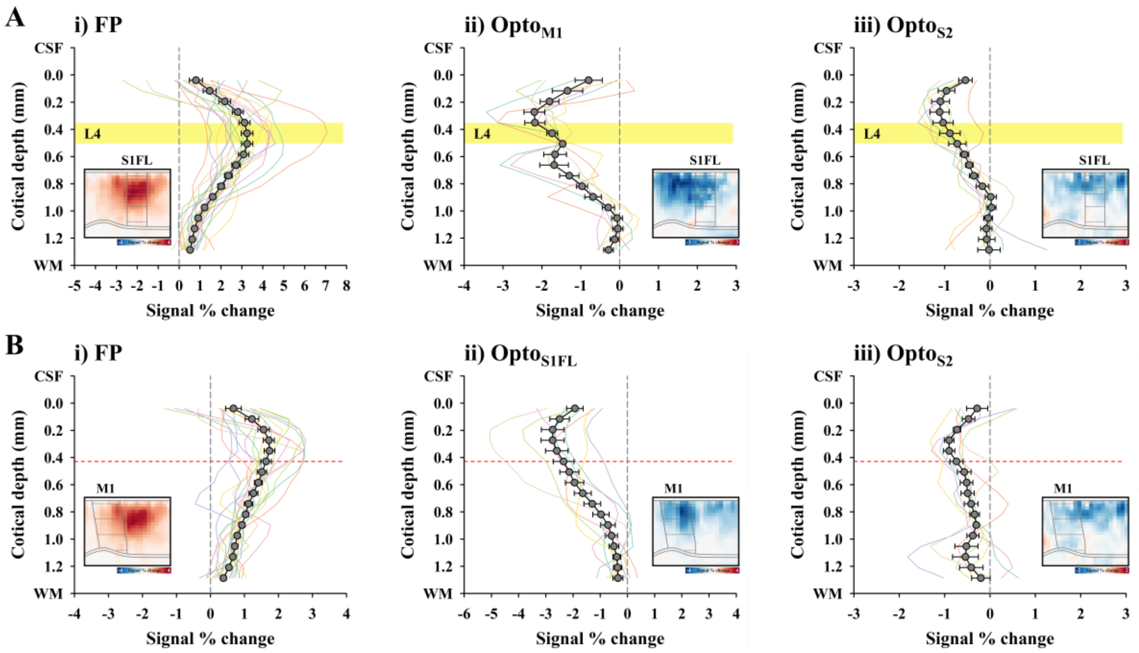

Figure S9. Cortical depth-dependent CBV responses of individual animals in the S1FL and M1 during forepaw stimulation and optogenetic cortical inactivation.

CBV responses in **(A)** the S1FL and **(B)** M1 of individual animals during forepaw stimulation and optogenetic cortical inactivation were plotted as a function of cortical depth. Inset images, averaged cortical-flattened fMRI maps; profiles with black circles, animal-wise averaged response; yellow-dashed box in A, ~150 µm width position of L4 of the S1FL; red-dashed line in B, laminar border of L2/3 and L5 in M1.

Although there was a large inter-animal variation, two peaks were observed in the S1FL when M1 was suppressed. When one animal profile with two strong peaks (green profile) was removed, the averaged profile of the remaining six animals also showed two peaks, indicating that the two peaks were indeed genuine and that the CBV point spread function was less than 150 µm FWHM.

Table S1. Resting-state functional connectivity of the somatosensory network measured by resting-state fMRI and cortical inactivation fMRI.

ROI & Study \ Downstream ROI		Ipsilateral hemisphere					Contralateral hemisphere				
		**S1FL**	**M1**	**S2**	**VPL**	**POm**	**S1FL**	**M1**	**S2**	**VPL**	**POm**
**S1FL**	**rs-fMRI (Z)**	**Seed-ROI**	0.42±0.04	0.32±0.03	-0.12±0.04	-0.02±0.02	0.35±0.03	0.31±0.04	0.23±0.03	-0.07±0.03	-0.00±0.03
	**Inactivation (%)**	**-1.41±0.19**	-1.34±0.17	-1.03±0.07	-0.65±0.05	-1.29±0.11	-0.31±0.03	-0.21±0.05	-0.05±0.05	0.09±0.08	0.10±0.11
**M1**	**rs-fMRI (Z)**	0.42±0.04	**Seed-ROI**	0.25±0.03	-0.12±0.03	0.00±0.02	0.31±0.03	0.38±0.05	0.21±0.03	-0.13±0.04	-0.00±0.01
	**Inactivation (%)**	-1.38±0.12	**-1.29±0.15**	-0.71±0.06	-0.33±0.08	-1.29±0.17	-0.28±0.07	-0.45±0.10	-0.08±0.09	-0.04±0.15	0.13±0.05
**S2**	**rs-fMRI (Z)**	0.32±0.03	0.25±0.03	**Seed-ROI**	-0.15±0.04	-0.06±0.01	0.23±0.02	0.20±0.03	0.29±0.03	-0.09±0.04	0.01±0.01
	**Inactivation (%)**	-0.73±0.04	-0.35±0.03	**-1.04±0.08**	-0.13±0.05	-0.50±0.07	-0.11±0.05	-0.11±0.05	-0.31±0.03	-0.06±0.06	0.03±0.22

The mean ± SEM is reported for resting-state fMRI (rs-fMRI, wild-type, 10 dataset from n = 5), S1FL inactivation (VGAT-ChR2, n = 8), M1 inactivation (VGAT-ChR2, n =7) and S2 inactivation (VGAT-ChR2, n = 7). The strengths of functional connectivity in rs-fMRI were calculated as the synchronization of resting fluctuations (z-score) with the seed-ROI area, whereas the spontaneous connectivity in cortical silencing fMRI was described as regional signal changes during optogenetic inactivation.

Table S2. Multiunit activity characteristics of somatosensory-driven long-range input and local recurrent activity in the S1FL.

Study / Measurement	Total somatosensory activity	Somatosensory input activity	Local recurrent activity
**Onset latency (ms)**	14.83 ± 1.22	14.50 ± 1.67	20.00 ± 1.21**,##
**Peak latency (ms)**	30.83 ± 1.40	26.50 ± 1.43**	32.83 ± 1.45**,##
**FWHM**	14.41 ± 0.88	12.36 ± 0.94**	12.55 ± 0.96*
**Peak amplitude (spike/ms)**	0.79 ± 0.11	0.37 ± 0.07**	0.59 ± 0.90*
**∑MUA (spike/ms)**	11.71 ± 1.34	4.55 ± 0.81**	7.45 ± 0.92**
**Goodness of fit (R^2)**	> 0.99	> 0.99	> 0.99

The mean ± SEM is reported for the 16-channel averaged multiunit activity (MUA) of the total somatosensory activity, input activity, and local recurrent activity (VGAT-ChR2, n = 6). The dynamic properties, including latencies (times to onset and peak of response) and full width at half-maximum (FWHM), and the MUA response, including peak amplitude and ∑MUA, were determined from the MUA trace fitted with a single Gaussian function. Onset latency was defined as the time for the first bin of five continuous bins whose spike rate differed significantly from the prestimulus baseline spike rate ($p < 0.05$, one-sample t-test). The goodness-of-fit between the single Gaussian function and the MUA trace is presented as the R^2 value. *$p < 0.05$ and **$p < 0.01$ for vs. total somatosensory activity, $^{\#}p < 0.05$ and $^{\#\#}p < 0.01$ for vs. somatosensory input activity (one-way analysis of variance with repeated measures followed by the Bonferroni *post hoc* test).

Table S3. Local field potential characteristics of somatosensory-driven long-range input and local recurrent activity in the S1FL.

Measurement \ Study	Total somatosensory activity	Somatosensory input activity	Local recurrent activity
**Peak latency (ms)**	36.00 ± 1.77	31.67 ± 1.52**	38.00 ± 1.71**,##
**FWHM**	20.62 ± 1.21	19.38 ± 1.12**	18.59 ± 1.62*
**Peak amplitude (mv)**	-0.69 ± 0.10	-0.28 ± 0.05*	-0.46 ± 0.09*
**∑LFP (mv)**	-23.10 ± 3.30	-7.36 ± 1.31*	-16.30 ± 3.34*
**Goodness of fit (R^2)**	> 0.99	0.99 ± 0.01	0.99 ± 0.00

The mean ± SEM is reported for the 16-channel averaged local field potential (LFP) of the total somatosensory activity, input activity, and local recurrent activity (VGAT-ChR2, n = 6). The dynamic properties, including peak latency and full width at half-maximum (FWHM), and the MUA response, including peak amplitude and ∑LFP, were determined from the LFP waveform fitted with a double gamma variate function. The goodness-of-fit between the gamma variate function and the LFP waveform is presented as the R^2 value. *$p < 0.05$ and **$p < 0.01$ for vs. total somatosensory activity, #$p < 0.05$ and ##$p < 0.01$ for vs. somatosensory input activity (one-way analysis of variance with repeated measures followed by the Bonferroni *post hoc* test).

Dataset S1 (separate file). Brain-wide spontaneous and evoked responses of somatosensory networks measured by CBV-weighted fMRI with cortical silencing and electrophysiological recording.

SI References

1. F. Schlegel *et al.*, Fiber-optic implant for simultaneous fluorescence-based calcium recordings and BOLD fMRI in mice. *Nature protocols* **13**, 840-855 (2018).
2. H. J. Shim *et al.*, Mouse fMRI under ketamine and xylazine anesthesia: Robust contralateral somatosensory cortex activation in response to forepaw stimulation. *NeuroImage* **177**, 30-44 (2018).
3. W. B. Jung, H. J. Shim, S. G. Kim, Mouse BOLD fMRI at ultrahigh field detects somatosensory networks including thalamic nuclei. *NeuroImage* **195**, 203-214 (2019).
4. H. J. Shim, J. Lee, S. G. Kim, BOLD fMRI and hemodynamic responses to somatosensory stimulation in anesthetized mice: spontaneous breathing vs. mechanical ventilation. *NMR in biomedicine* **33**, e4311 (2020).
5. B. A. Duffy, M. Choy, M. R. Chuapoco, M. Madsen, J. H. Lee, MRI compatible optrodes for simultaneous LFP and optogenetic fMRI investigation of seizure-like afterdischarges. *NeuroImage* **123**, 173-184 (2015).
6. D. Bernal-Casas, H. J. Lee, A. J. Weitz, J. H. Lee, Studying Brain Circuit Function with Dynamic Causal Modeling for Optogenetic fMRI. *Neuron* **93**, 522-532.e525 (2017).
7. S. G. Kim *et al.*, Cerebral blood volume MRI with intravascular superparamagnetic iron oxide nanoparticles. *NMR in biomedicine* **26**, 949-962 (2013).
8. H. G. Rey, C. Pedreira, R. Quian Quiroga, Past, present and future of spike sorting techniques. *Brain research bulletin* **119**, 106-117 (2015).
9. H. S. Moon *et al.*, Contribution of Excitatory and Inhibitory Neuronal Activity to BOLD fMRI. *Cerebral cortex (New York, N.Y. : 1991)* **31**, 4053-4067 (2021).
10. K. H. Pettersen, A. Devor, I. Ulbert, A. M. Dale, G. T. Einevoll, Current-source density estimation based on inversion of electrostatic forward solution: effects of finite extent of neuronal activity and conductivity discontinuities. *Journal of neuroscience methods* **154**, 116-133 (2006).
11. R. W. Cox, AFNI: software for analysis and visualization of functional magnetic resonance neuroimages. *Computers and biomedical research, an international journal* **29**, 162-173 (1996).
12. S. M. Smith *et al.*, Advances in functional and structural MR image analysis and implementation as FSL. *NeuroImage* **23 Suppl 1**, S208-219 (2004).
13. B. B. Avants *et al.*, A reproducible evaluation of ANTs similarity metric performance in brain image registration. *NeuroImage* **54**, 2033-2044 (2011).
14. W. B. Jung, G. H. Im, H. Jiang, S.-G. Kim, Early fMRI responses to somatosensory and optogenetic stimulation reflect neural information flow. *Proceedings of the National Academy of Sciences* **118**, e2023265118 (2021).

15. P. K. Mandal, R. Mahajan, I. D. Dinov, Structural brain atlases: design, rationale, and applications in normal and pathological cohorts. *Journal of Alzheimer's disease : JAD* **31 Suppl 3**, S169-188 (2012).
16. S. W. Oh *et al.*, A mesoscale connectome of the mouse brain. *Nature* **508**, 207-214 (2014).
17. M. D. Fox, M. E. Raichle, Spontaneous fluctuations in brain activity observed with functional magnetic resonance imaging. *Nature reviews. Neuroscience* **8**, 700-711 (2007).
18. Y. Huo, H. Chen, Z. V. Guo, Mapping Functional Connectivity from the Dorsal Cortex to the Thalamus. *Neuron* **107**, 1080-1094.e1085 (2020).
19. Y. Y. Shih *et al.*, Ultra high-resolution fMRI and electrophysiology of the rat primary somatosensory cortex. *NeuroImage* **73**, 113-120 (2013).
20. S. Zhao *et al.*, Cell type–specific channelrhodopsin-2 transgenic mice for optogenetic dissection of neural circuitry function. *Nature methods* **8**, 745-752 (2011).
